# Supplementary material for: Efficacy of smartphone-based virtual reality relaxation in providing comfort to patients with cancer undergoing chemotherapy in oncology outpatient setting in Indonesia: protocol for a randomised controlled trial
Source: BMJ Open. 2023 Jul 25;13(7):e074506. doi: 10.1136/bmjopen-2023-074506 (PMC10373714; doi:10.1136/bmjopen-2023-074506)
Supplement: Author's manuscript [file bmjopen-2023-074506.draft_revisions.pdf]

BMJ Open is committed to open peer review. As part of this commitment we make the peer review history of every article we publish publicly available.

When an article is published we post the peer reviewers' comments and the authors' responses online. We also post the versions of the paper that were used during peer review. These are the versions that the peer review comments apply to.

The versions of the paper that follow are the versions that were submitted during the peer review process. They are not the versions of record or the final published versions. They should not be cited or distributed as the published version of this manuscript.

BMJ Open is an open access journal and the full, final, typeset and author-corrected version of record of the manuscript is available on our site with no access controls, subscription charges or pay-per-view fees (http://bmjopen.bmj.com).

If you have any questions on BMJ Open's open peer review process please email info.bmjopen@bmj.com

BMJ Open

Efficacy of Smartphone-Based Virtual Reality Relaxation in Providing Comfort to Patients with Cancer Undergoing Chemotherapy: Protocol for a Randomized Controlled Trial

Journal:	*BMJ Open*
Manuscript ID	bmjopen-2023-074506
Article Type:	Protocol
Date Submitted by the Author:	10-Apr-2023
Complete List of Authors:	Gautama, Made Satya Nugraha ; Taipei Medical University; Universitas Gadjah Mada<br>Haryani, Haryani; Universitas Gadjah Mada<br>Huang, Tsai-Wei ; Taipei Medical University, School of Nursing, College of Nursing; Taipei Municipal Wan-Fang Hospital, Research Center in Nursing Clinical Practice, Department of Nursing
Keywords:	Virtual Reality, CHEMOTHERAPY, Nursing Care, Adult palliative care < PALLIATIVE CARE

SCHOLARONE™
Manuscripts

Efficacy of Smartphone-Based Virtual Reality Relaxation in Providing Comfort to Patients with Cancer Undergoing Chemotherapy: Protocol for a Randomized Controlled Trial

XX,[1,2] XX,[3] XX[1,4,5,6]

[1]School of Nursing, College of Nursing, Taipei Medical University, Taipei, Taiwan

[2]Master of Nursing Program, Faculty of Medicine Public Health and Nursing, Universitas Gadjah Mada, Yogyakarta, Indonesia

[3]Department of Surgical Medical Nursing, Faculty of Medicine, Public Health and Nursing, Universitas Gadjah Mada, Yogyakarta, Indonesia

[4]Research Center in Nursing Clinical Practice, Department of Nursing, Wan Fang Hospital, Taipei Medical University, Taipei, Taiwan

[5]Department of Nursing, Wan Fang Hospital, Taipei Medical University, Taipei, Taiwan

[6]Cochrane Taiwan, Taipei Medical University, Taipei, Taiwan

***Corresponding Author:**

XX

For peer review only

Short running head: Smartphone-Based Virtual Reality Relaxation for Comfort in Chemotherapy

Data Availability Statement

All data reported in this manuscript can be found in the literature cited in the text.

Conflicts of Interest

The authors have no conflicts of interest to disclose.

Ethics Approval and Consent to Participate

The study was conducted in accordance with the Declaration of Helsinki (as revised in 2013). Ethics approval was obtained on XX. The protocol was approved at the ClinicalTrial.gov, Registry of Clinical Trials on 2023/03/04 (XXX). Any modification to the protocol will be approved by the ethics committee prior to its implementation. All participants will sign a written informed consent form.

Consent for publication

The participant consent forms will be made available upon request to the corresponding author.

Acknowledgement

This manuscript was edited by Wallace Academic Editing.

For peer review only

Authors' Contributions

SNG, HH, and TWH conceptualized and designed the study. TWH and HH provided methodological advice related to the study design. The manuscript was written by SNG and TWH and reviewed by all authors. All authors designed the statistical analysis. All authors made critical revisions to the manuscript and provided final approval for the manuscript.

Funding

This research did not receive external funding. The APC was funded by Wan Fang Hospital, grant number 111-wf-f-5.

For peer review only

Abstract

Introduction: Patients with cancer undergoing chemotherapy experience various physical and psychological problems and discomfort. Virtual reality (VR) can be used in technology-based nonpharmacological therapy that can serve as a potential distractor in the symptom management of patients with cancer undergoing chemotherapy. We propose a Smartphone-Based Virtual Reality Relaxation (S-VR) technique as a complementary modality to provide comfort to patients with cancer, and we will evaluate its effect on patients with cancer undergoing chemotherapy.

Methods and analysis: We will recruit 80 patients from the One Day Chemotherapy "Tulip" Unit of Dr. Sardjito General Hospital, Yogyakarta, Indonesia. This will be a two-arm parallel randomized controlled trial, with a 1:1 allocation and the primary outcome assessor blinded. This study will be divided into two group: (2) an intervention group, with the participants receiving 360° panoramic video content and music relaxation intervention through a VR device (head-mounted display) placed on their head during chemotherapy for ±10 minutes plus standard care, and (2) a control group, with the participants receiving guided imagery relaxation therapy in the form of leaflet plus standard care. We will measure the outcomes after one chemotherapy cycle for each participant. The primary outcome is the effectiveness of the S-VR in improving the comfort of patients. The secondary outcome is the effect of the S-VR on the patients' symptom management self-efficacy, pain, anxiety, blood pressure (SBP and DBP), and pulse rate.

Ethics and dissemination: This study was approved by the Medical and Health Research Ethics Committee (MHREC) of the Faculty of Medicine, Public Health and Nursing of Universitas Gadjah Mada—Dr. Sardjito General Hospital, Yogyakarta, Institutional Review Board (IRB) (Approval number: KE/FK/0301/EC/2023). Written informed consent will be obtained from all

For peer review only

participants who enroll in the study. Dissemination will be conducted through peer-reviewed publications and conference presentations.

Trial Registration Number: NCT05756465

Keywords: Virtual Reality; chemotherapy; relaxation; comfort; self-efficacy

For peer review only

Strengths and Limitations of This Study

- This study proposes a sophisticated, novel approach to using immersive virtual reality as a nonpharmacological intervention to alleviate discomfort in adult patients with cancer who are undergoing chemotherapy.
- The intervention involves the administration of 360° videos made by the researchers and that have natural panoramic views integrated with relaxation music and can be easily accessed using smartphones with Internet through VR devices (head-mounted displays) in both hospital and home settings. The operation of the tools involved and accessing the content does not require specialized knowledge.
- We will identify potential confounding factors that may cause bias by using multivariate statistical analysis.
- We will measure the effect of the S-VR intervention only over a short period (1 cycle of chemotherapy) without user feedback or long-term follow-up.

INTRODUCTION

The cancer incidence in Indonesia (136.2/100,000 population) is the 8th highest in Southeast Asia and 23rd highest in Asia.[1] According to Basic Health Surveillance (Riskesdas),[2] the prevalence of cancer in Indonesia increased from 1.4 in 2013 to 1.49 in 2018. Of the total of 396,914 cancer cases in Indonesia in 2020, the top three most common cancer types were breast cancer (65,858 cases; 16.6%), cervical cancer (36,633 cases; 9.2%), and lung cancer (21,392 cases; 8.8%).[3] The region with the highest prevalence of cancer is the Special Region of Yogyakarta Province (4.86/1000 population), and 23% of the patients with cancer in this region receive chemotherapy, which is the second-most-common cancer management strategy after surgery.[2]

Patients undergoing chemotherapy experience various adverse effects.[4] Moreover, they may experience physical and psychological discomfort caused by the treatment drugs, invasive procedures, and chemotherapy environments. In general, 86% of patients with cancer experience pain, nausea, vomiting, mucositis, diarrhea, and alopecia.[5 6] Other side effects include psychological problems, such as anxiety, feelings of hopelessness, depression, anger, fear, vulnerability, and a sense of loss of control.[7] Management of the side effects of chemotherapy by using drugs is not fully effective and may cause other side effects,[8] which can cause further patient discomfort.[9 10]

Comfort is defined as a lack of pain, distress, worry, and uneasiness, and it is one of the most crucial factors in holistic nursing care.[11 12] Focusing on patients' biophysiological aspects (e.g., symptom management) as well as psychospiritual, environmental, and sociocultural aspects related to healing is crucial for maintaining their well-being.[13] Kolcaba explained that in addition to providing basic care to patients, nurses must provide special and unique nursing care to each

For peer review only

patient to increase patient comfort.[14 15] Patients with cancer require physical, social, and spiritual support and a comfortable environment to have a sense of life meaning.[13 16 17] Meeting the comfort needs of patients with cancer positively affects the patients' satisfaction with the services they receive, their treatment adherence, and their overall quality of life.[12 18]

Many nonpharmacological or alternative approaches are used in the management of chemotherapy side effects.[19] Some studies have reported that nonpharmacological therapies, such as music therapy, acupuncture, massage, guided imagery, hypnosis, acupressure, and virtual reality (VR) therapy, can alleviate physical and psychological symptoms in patients with cancer.[20 21] However, most nonpharmacological therapies involve the use of conventional methods; few have incorporated technologies, such as VR, into alternative therapy.[22] The use of VR in both cancer research and therapy remains limited.[23]

VR has gained in popularity as a tool for alternative therapy because it can overcome some of the limitations of traditional therapy.[24 25] VR is widely used in cancer care, especially to reduce patient anxiety and pain during invasive procedures.[22 26] The immersive experience of VR provides visual and auditory stimuli in a virtual environment, which has the therapeutic effect of reducing patient anxiety and pain.[27] VR has been demonstrated to effectively reduce anxiety, pain, fatigue, and stress and to lead to positive outcomes in patients undergoing chemotherapy.[21 28 29] In addition to reducing pain, VR therapy can increase patient comfort during treatment in the ICU.[30]

Although scientific evidence of the effectiveness of using VR in the treatment of cancer continues to grow, findings regarding the use of smartphone-integrated VR remain limited.[31 32] Smartphone-based VR is not only cost-effective, simple, portable, and immersive but also

For peer review only

effective in providing positive experiences, reducing negative emotions, and managing pain in patients with cancer.[33 34] Such VR therapy integrates visual stimulation and relaxation audio from smartphones or tablets and can be used as an alternative therapy to manage the symptoms of patients undergoing chemotherapy by reducing anxiety and distracting patients from pain.[22 32 35] Increased comfort positively affects the satisfaction, acceptance of medication side effects, and quality of life of patients with cancer.[20 36] Therefore, in the present study, we proposed an innovative strategy for the management of discomfort in patients undergoing chemotherapy by using smartphone-based VR relaxation (S-VR) with content including natural panoramic views and three-dimensional relaxation music.

The primary objective of this study is to determine the efficacy of S-VR in increasing comfort in patients with cancer undergoing chemotherapy. The secondary objectives of this study are to assess the effects of S-VR on the symptom management self-efficacy, anxiety, pain, and vital signs (pulse and blood pressure) of patients with cancer undergoing chemotherapy. We hypothesize that relative to the control group, the patient group that receives the S-VR intervention will experience greater improvements in their comfort levels.

For peer review only

METHODS AND ANALYSIS

Design

This study is a two-arm parallel randomized controlled trial with a 1:1 allocation. The study will divide the patients into two groups: the intervention group, in which the patients will receive S-VR therapy through a VR device (head-mounted display) mounted on the patients' heads during chemotherapy plus standard care (daily routine care in administration of chemotherapy drugs), and the control group, in which the patients will receive standard care and guided imagery therapy with leaflets containing instructions for techniques for relaxing through image visualization. The group allocation will be implemented using the envelope concealment method, and the outcome assessor will be blinded to the group allocation during data collection. The protocol has been designed in accordance with the Standard Protocol Items: Recommendations for Interventional Trials and its checklist.[37 38] Figure 1 presents the modified CONSORT (2017) flow diagram.[39]

Setting

The Special Region of Yogyakarta (DIY) Province will be the study location because it has the highest prevalence of cancer in Indonesia. The national prevalence of cancer is 1.79/1000 population, whereas the prevalence in the DIY Province is 4.86/1000 population, with 23% of these patients undergoing chemotherapy treatment.[2] This study will be conducted at the One Day Chemotherapy Polyclinic "Tulip" Integrated Cancer Unit of Dr. Sardjito General Hospital, Yogyakarta, Indonesia. According to the annual report of the service performance of the Integrated Cancer Unit of Dr. Sardjito General Hospital, the number of visits by patients with cancer was 100,614 people in 2019, an increase of 19.8% from the previous year.

For peer review only

Patient and public involvement

The protocol has been designed in close collaboration between the School of Nursing, Taipei Medical University, and the Master of Nursing Programme, Universias Gadjah Mada, with strong support from Dr. Sardjito General Hospital, Yogyakarta. We work with cancer patients undergoing chemotherapy under the Integrated Cancer Unit at Dr. Sardjito General Hospital Yogyakarta, Indonesia. A patient with cancer undergoing chemotherapy was enrolled in this intervention programme and completed the study evaluation. Valuable input, preference, and evaluation on the part of both patients and hospital institutions have been invaluable in the development of this intervention, thus, we hope that the findings from this RCT will be disseminated to local, regional, and national policymakers as well as an international scientific audience.

Population and Sample

The target population is patients with cancer who will undergo chemotherapy at the "Tulip" Integrated Cancer Unit of Dr. Sardjito General Hospital. The inclusion criteria for this study are as follows:

1. Being a patient with cancer receiving chemotherapy in all cycles
2. Being aged ≥18 years
3. Having a performance status (ECOG Score) of 0–2
4. Being able to understand and sign the informed consent form

Respondents who meet the following exclusion criteria will be excluded from the study:

For peer review only

1. Being a patient with cancer with a history of skull structure or cervical spine abnormalities that may complicate the use of VR devices
2. Being a cancer patient with cognitive, visual, and hearing impairment; wearing glasses; or having a chemo port
3. Being a patient with a history of epilepsy, seizures, drug or drug addiction, vertigo, or visually induced motion sickness

Sample Size Calculation

The sample size was calculated using free-to use software, namely G*Power for Windows Version 3.1.9.6.[40] The effect size is 0.81, type 1 error (α) = 5% (two-tailed), power (1-β) 90%, and the allocation ratio between the groups is 1:1. The effect size was set with reference to previous studies with similar outcomes.[10] According to the G*Power calculation, the study sample size should be 34 per group. With consideration of an expected dropout rate of 15%, the sample size was increased to 40 per group.

The participant dropout criteria are as follows:

1. The patient being unwilling to continue the sequence of the study process (lost to follow-up)
2. The patients experiencing drug extravasation incidents or severe side effects during chemotherapy

The consecutive sampling technique will be applied. This nonprobability sampling method involves recruiting all individuals from the accessible population during a given time interval or

For peer review only

for a specific sample size. Patients with cancer who have scheduled chemotherapy and meet the requirements of the data collection time will be asked to participate in the study.

Participant Characteristics and Study Outcomes

Participant characteristics

A questionnaire will be used to collect patient demographic data, including age, sex, education level, marital status, occupation, income, and assistance during chemotherapy, and clinical characteristics, including the type of cancer, body surface area (height and weight), cancer stage, duration of diagnosis, chemotherapy cycle, and ECOG score.

Outcomes

The primary and secondary outcomes are summarized in Table 1.

Comfort of cancer patients is measured using the shortened general comfort questionnaire (SGCQ) instrument by Chatarine Kolcaba in 2006 which has been tested for validity and reliability in Indonesian version by Artanti et al.[41] The validity test reported that Content validity index (CVI) of item (I-CVI) and scale (S-CVI) score were both 1 (valid). Cronbach's alpha score showed 0.769, meaning that the SGCQ Indonesian version is reliable. The SGCQ instrument describes individual comfort by assigning a Likert score to 28 items. Likert scores range from 1 (strongly disagree) to 6 (strongly agree). The highest total score is 168, and the lowest is 28.

Self – efficacy is measured by using the Self-Efficacy of Cancer Symptom Self-Management Scale (SE – CSSMS),[42] which consists of 6 question items with a value range of 1 (not at all sure) to 10 (very sure). SE - CSSMS has been tested for validity and reliability on cancer patients in Indonesia.[42] The content validity index (CVI) is 0.94. The r-value is 0.467, which is greater

For peer review only

than the r-table value of 0.12, so these results state that the six items in the symptom management self-efficacy instrument are valid. The reliability test results showed that the Cronbach alpha value is 0.90 (> 0.70), so the measuring instrument is reliable.

Anxiety is measured using the Visual Analog Scale for Anxiety (VAS – A) in the form of a horizontal line with a scale of 100 millimeter or 10 centimeters, with the left by the words "not at all anxious" and on the right by "extremely anxious".[43 44] VAS-A has a sensitivity of 76.8%, specifications of 64.9%, and internal reliability consistency (Cronbach Alpha ≥0.7.[43]

Pain is measured using the numeric rating scale (NRS). The NRS consist of numerical scale; from 0 to 100 (101 – point scale), 0 to 10 (11 – point scale) or 1 to 10 (10 – point scale). This study will use a 0 – 10 NRS due to it provides more sufficient sensitivity to measure pain in most cancer patients.[45] The NRS has been declared valid and reliable for subjective measurement of cancer pain, both due to adverse effects of cytotoxic agents, cancer treatment procedures (surgery, radiotherapy, and chemotherapy), and the malignancy of the cancer itself.[46]

Pulse rate and blood pressure (systolic and diastolic pressure) are measured using an automatic digital sphygmomanometer. According American Heart Association, the regular resting heart rate for adults is 60 to 100 beats per minute, and systolic blood pressure is above 90 mm Hg and less than 120 mm Hg, with a diastolic pressure between 60 mm Hg and less than 80 mm Hg.[47]

Comfort and self-efficacy for symptom management will be measured at two time points: at baseline (before chemotherapy premedication) and posttest (after the chemotherapy session is completed). Anxiety, pain, and vital signs (pulse rate and blood pressure) will be measured at five time points: thrice at pretest (baseline data collection, before the installation of intravenous access, and before administration of VR intervention) and twice at posttest (immediately after

For peer review only

intervention and after the chemotherapy session is completed). The timeline of the data collection and measurement is presented in Table 2.

Randomization and Concealment

The researchers will screen to identify patients who are eligible to participate in the study. The patients' medical records will be evaluated to verify their baseline information (e.g., anthropometric data, laboratory data, patient identification data, and laboratory results). An allocation sequence will be generated through simple randomization of the study participants by using a formula on the Microsoft Excel program (the RANDBETWEEN function), and the allocation sequence will be concealed in sealed envelopes. Thereafter, the participants in the intervention and control groups will receive envelopes containing random numbers generated using the randomization procedure. The outcome assessor (the nurse appointed by the researcher to be responsible for outcome data collection) and data analysts will be blinded to the participant allocation. Due to the technical nature of the VR interventions; it was difficult to blind the study participants. Thus, to minimize potential methodological bias, the outcome assessor and data analysts will be blinded.[48]

For peer review only

Patient Recruitment

Patients who are scheduled for chemotherapy will be assessed by the nurse on duty, who will review their latest laboratory results, medical records, vital signs, and electrocardiogram results, to determine whether they are eligible to undergo chemotherapy. The patients who pass the screening process will be asked whether they are willing to be involved in the study and will be asked to sign the informed consent form. Patients who are willing and who sign the informed consent form will be given a sealed envelope containing a random number indicating their group

allocation. The participants will be evaluated (pretest) by the outcome assessor (research nurse) before they enter the chemotherapy room. After the evaluation, the researcher will open each participant's envelope and see the random number that they have been assigned (without the knowledge of the participants or the outcome assessor). If the number assigned is between 1 and 40, the participant will be directed to the chemotherapy room on the east side of the hospital (intervention group); if the number is between 41 and 80, the participant will be directed to the chemotherapy room on the west side of the hospital (control group). We will for the participants in the intervention and control groups to be directed to different chemotherapy rooms, and a research assistant (RA) will accompany the participants to the rooms to minimize contamination of information between participants, between participants and intervention providers and between the nurses or doctors on duty.[49]

Intervention (S-VR + standard care)

Participants in the intervention arm will receive immersive VR intervention with a VR box (Shinecon 6.0 VR Box Virtual Reality Glasses with headphones). S-VR is a virtual relaxation and distraction therapy administered through a smartphone-based VR device (head-mounted display) mounted to the head of the patient with cancer during chemotherapy. The intervention involves a virtual environment in which 360° videos of natural panoramas with traditional and classical music (noncopyright) are provided. The S-VR device is paired with a smartphone with either an Android (minimum generation 11, Android 4.4 KitKat) or iOS (version 12, minimum screen size of 5.5 inch and maximum of 6.0 inch, screen resolution of at least 1080 × 1920 pixels) operating system and the ability to connect to WIFI or the Internet and with the YouTube app installed, which they will use to access the 360° videos. The S-VR content comprises original 360° videos approximately 10 minutes long and produced by the researchers by using

For peer review only

video editing software. The video duration was determined on the basis of a previous study by Fabi et al.[50] in which this duration was demonstrated to be effective in preventing patient saturation related to content and preventing motion sickness. The 360° videos present panoramic views of Yogyakarta, Indonesia, and Taipei, Taiwan (Figure 2). The RA will provide information about the S-VR intervention for approximately 5 minutes, assist in attaching the S-VR devices to the patient, and conduct the intervention for approximately 10 minutes.

Control (Leaflet + standard care)

In the control group, participants will be provided with standard care and guided imagery leaflets, which will provide information on the meaning, benefits, and methods of completing guided imagery relaxation therapy during chemotherapy. The RA will guide the participants through the practices of the guided imagery relaxation therapy described in the leaflet for ±10 minutes (Figure 3).

Compensation

Compensation will be provided by the researchers to all respondents after the chemotherapy session in the form of gifts of appreciation of their participation in this research.

Safety and Side Effect Management

The intervention of this study is not associated with any significant side effects because it does not involve invasive procedures and is administered for a short period (1 chemotherapy cycle). However, we predict that the participants will feel discomfort both while using the VR and during chemotherapy and will record their levels of discomfort on a monitoring sheet. During the

For peer review only

study, the researchers will clearly indicate that they can be contacted should the participants have any problems or complaints or if something unexpected occurs.

Data Analysis

The collected data will be processed using IBM SPSS statistical software version 23 for Windows through editing, coding, and data entry processes. The statistician will be blinded to the study outcomes and will conduct analyses on the basis of the labeling of the groups. For the treatment group, the demographic and clinical variables recorded at enrollment will be tabulated. The descriptive statistics will be presented as numbers, means, standard deviations, medians, minimums, and maximums. Homogeneity and normality tests will be conducted before the bivariate analysis test. A homogeneity test for categorical data (age, sex, education level, marital status, occupation, income, the presence of a family member or caregiver during chemotherapy, type of cancer, stage of cancer, duration of cancer diagnosis, chemotherapy cycle, and ECOG scores) will be performed using the chi-square or Fisher's test, whereas a homogeneity test for numerical data (height and weight) will be performed using Levene's test if the data are normally distributed and using the Mann–Whitney U test if the data are not normally distributed. Both groups will be considered to be homogeneous if $p > 0.05$. Bivariate analysis will be used to determine the differences in the mean scores for comfort level and symptom management self-efficacy before and after treatment in the intervention and control groups. If the data distribution is normal, we will use a paired t test and independent t test. However, if the data distribution is not normal, the nonparametric Wilcoxon and Mann–Whitney U tests will be used if significant differences are identified ($p < 0.05$). For anxiety, pain, and vital-sign (blood pressure and pulse rate) outcome measurement analysis, General Linear Model-Repeated Measure (GLM-RM) statistics will be used. The GLM-RM is a statistical test used for the comparative analysis of two

For peer review only - http://bmjopen.bmj.com/site/about/guidelines.xhtml

unpaired numerical data groups with more than two measurements and is applied when the effect of a treatment or intervention is measured at different time points. If the data are not normally distributed, the repeated Mann–Whitney alternative test with Bonferroni correction will be used. The present study will use statistical method to adjust for potentially confounding effects of the S-VR intervention on the patient's outcomes. Multivariate model can handle identified confounders and minimize the bias effect of the intervention.[51] Linear regression analysis will be applied by using the enter method. Adjustment by using this analysis can clarify that how much the confounders in the model distort the intervention and outcome.[52]

Ethics and Dissemination

The present study was approved by the Medical and Health Research Ethics Committee (MHREC) Faculty of Medicine, Public Health and Nursing Universitas Gadjah Mada—Dr. Sardjito General Hospital, Yogyakarta, IRB (Approval number: KE/FK/0301/EC/2023). All participants enrolled in the study will be asked to provide written informed consent, which will include a statement requesting their willingness to allow the data to be share. All information related to the identity of the participants will be kept confidential by the researchers. No posttrial care has been planned. The study results will be published in a peer-reviewed scientific journal. The results will not indicate the identity of any participant. Authorship eligibility was based on an individual making substantial contributions to the study and manuscript review. Access to the final data set will be granted to the primary investigator and coinvestigators. The study protocol is available on clinicaltrials.gov (ClinicalTrials.gov Identifier: NCT05756465). The study is scheduled to start recruitment at Dr. Sardjito General Hospital in Indonesia in April 2023. After the implementation of the intervention, data collection, and statistical analysis, we expect to submit the results to a peer-reviewed journal in October 2023.

For peer review only

For peer review only

Contributors

SNG developed the initial trial concepts and drafted the manuscript. TWH and HH contributed to the development of the manuscript. All authors have critically revised the manuscript for essential intellectual content. All authors have read and approved the submitted version of the manuscript.

Funding

This research did not receive external funding. The APC was funded by Wan Fang Hospital, grant number 111-wf-f-5.

Competing Interests

None to declare.

Patient and Public Involvement

Patients and the public will not be involved in the design, performance, reporting, or dissemination of this study.

Patient Consent for Publication

Not required.

Provenance and Peer Review

Not commissioned; external peer review

For peer review only

REFERENCES

1. Ministry of Health Republic of Indonesia [Internet]. 2019. Available from: https://www.kemkes.go.id/article/view/19020100003/hari-kanker-sedunia-2019.html
2. Basic Health Survailance/Riset Kesehatan Dasar tahun 2018 [Internet]. 2018. Available from: http://repository.bkpk.kemkes.go.id/3514/1/Laporan Riskesdas 2018 Nasional.pdf
3. Globocan. New Global Cancer Data: Indonesia [Internet]. 2020. Available from: https://gco.iarc.fr/today/data/factsheets/populations/360-indonesia-fact-sheets.pdf
4. Thiagarajan M, Chan CMH, Fuang HG, Beng TS, Atiliyana MA, Yahaya NA. Symptom prevalence and related distress in cancer patients undergoing chemotherapy. Asian Pacific J Cancer Prev. 2016;17(1):171–6.
5. Pearce A, Haas M, Viney R, Pearson S-A, Haywood P, Brown C, et al. Incidence and severity of self-reported chemotherapy side effects in routine care: A prospective cohort study. PLoS One [Internet]. 2017 Oct 10;12(10):e0184360. Available from: https://doi.org/10.1371/journal.pone.0184360
6. Şahin ZA, Ergüney S. Effect on Symptom Management Education Receiving Patients of Chemotherapy. J Cancer Educ [Internet]. 2016;31(1):101–7. Available from: https://doi.org/10.1007/s13187-015-0801-8
7. American Cancer Society. Chemotherapy side effects | american cancer society [Internet]. 2023. Available from: https://www.cancer.org/treatment/treatments-and-side-effects/treatment-types/chemotherapy/chemotherapy-side-effects.html
8. Nurgali K, Jagoe RT, Abalo R. Editorial: Adverse Effects of Cancer Chemotherapy: Anything New to Improve Tolerance and Reduce Sequelae? [Internet]. Vol. 9, Frontiers in Pharmacology . 2018. Available from: https://www.frontiersin.org/articles/10.3389/fphar.2018.00245
9. Keim-Malpass J, Levine B, Danhauer SC, Avis NE. Work-related perceptions and quality of life among breast cancer survivors. Psychooncology. 2016 Jul;25(7):873–6.
10. Bilgiç Ş, Acaroğlu R. Effects of Listening to Music on the Comfort of Chemotherapy Patients. West J Nurs Res [Internet]. 2016 Aug 11;39(6):745–62. Available from: https://doi.org/10.1177/0193945916660527

For peer review only

11. Kolcaba KY. Holistic comfort: Operationalizing the construct as a nurse-sensitive outcome. Adv Nurs Sci [Internet]. 1992;15(1):1–10. Available from: https://www.researchgate.net/profile/Katharine-Kolcaba/publication/21635629_Holistic_comfort_operationalizing_the_construct_as_a_nurse-sensitive_outcome/links/54eb45810cf2082851bd7130/Holistic-comfort-operationalizing-the-construct-as-a-nurse-sensitive-ou
12. Can A, Hintistan S. An Assessment of the Comfort Level of Cancer Patients with Receiving Chemotheraphy. Bezmialem Sci. 2021;9(3):310–6.
13. Smith MC, Parker ME. Nursing Theories and Nursing Practice 4th ed. Philadelphia, PA: F.A. Davis; 2015.
14. Kolcaba KY. The Art of Comfort Care. Image J Nurs Scholarsh [Internet]. 1995 Dec 1;27(4):287–9. Available from: https://doi.org/10.1111/j.1547-5069.1995.tb00889.x
15. Nuraini T, Gayatri D, Rachmawati İ. Comfort assessment of cancer patient in palliative care: A nursing perspective. Int J Caring Sci [Internet]. 2017;10(1):209. Available from: https://internationaljournalofcaringsciences.org/docs/24_tuti_original_10_1.pdf
16. Kolcaba K. Comfort Theory and Practice: A Vision for Holistic Health Care and Research. New York, NY: Springer Publishing Company; 2003.
17. Cheng F, Wang W. Factors influencing comfort level in head and neck neoplasm patients receiving radiotherapy. Int J Nurs Sci [Internet]. 2014;1(4):394–9. Available from: https://www.sciencedirect.com/science/article/pii/S2352013214001021
18. Miao J, Liu M, Ma J, Wang H. Effectiveness of Comfort Nursing Combined with Continuous Nursing on Patients with Colorectal Cancer Chemotherapy. Wu X, editor. Evidence-Based Complement Altern Med [Internet]. 2022;2022:9647325. Available from: https://doi.org/10.1155/2022/9647325
19. Prigerson HG, Bao Y, Shah MA, Paulk ME, LeBlanc TW, Schneider BJ, et al. Chemotherapy Use, Performance Status, and Quality of Life at the End of Life. JAMA Oncol [Internet]. 2015 Sep 1;1(6):778–84. Available from: https://doi.org/10.1001/jamaoncol.2015.2378
20. Bani Mohammad E, Ahmad M. Virtual reality as a distraction technique for pain and anxiety among patients with breast cancer: A randomized control trial. Palliat Support Care [Internet]. 2018/09/10. 2019;17(1):29–34. Available from:

For peer review only

https://www.cambridge.org/core/article/virtual-reality-as-a-distraction-technique-for-pain-and-anxiety-among-patients-with-breast-cancer-a-randomized-control-trial/5487BEB7D104A20EF58EFA5432530B8C

21. Scates D, Dickinson JI, Sullivan K, Cline H, Balaraman R. Using Nature-Inspired Virtual Reality as a Distraction to Reduce Stress and Pain Among Cancer Patients. Environ Behav [Internet]. 2020 May 22;52(8):895–918. Available from: https://doi.org/10.1177/0013916520916259
22. Chirico A, Lucidi F, De Laurentiis M, Milanese C, Napoli A, Giordano A. Virtual Reality in Health System: Beyond Entertainment. A Mini-Review on the Efficacy of VR During Cancer Treatment. J Cell Physiol [Internet]. 2016 Feb 1;231(2):275–87. Available from: https://doi.org/10.1002/jcp.25117
23. Chirico A, Maiorano P, Indovina P, Milanese C, Giordano GG, Alivernini F, et al. Virtual reality and music therapy as distraction interventions to alleviate anxiety and improve mood states in breast cancer patients during chemotherapy. J Cell Physiol [Internet]. 2020 Jun 1;235(6):5353–62. Available from: https://doi.org/10.1002/jcp.29422
24. Stetz MAJ, Ries RI, Folen RA. Virtual reality supporting psychological health. In: Advanced Computational Intelligence Paradigms in Healthcare 6 Virtual Reality in Psychotherapy, Rehabilitation, and Assessment. Springer; 2011. p. 13–29.
25. Morina N, Ijntema H, Meyerbröker K, Emmelkamp PMG. Can virtual reality exposure therapy gains be generalized to real-life? A meta-analysis of studies applying behavioral assessments. Behav Res Ther [Internet]. 2015;74:18–24. Available from: https://www.sciencedirect.com/science/article/pii/S0005796715300334
26. Huang Q, Lin J, Han R, Peng C, Huang A. Using Virtual Reality Exposure Therapy in Pain Management: A Systematic Review and Meta-Analysis of Randomized Controlled Trials. Value Heal [Internet]. 2022;25(2):288–301. Available from: https://www.sciencedirect.com/science/article/pii/S1098301521015400
27. Chow H, Hon J, Chua W, Chuan A. Effect of Virtual Reality Therapy in Reducing Pain and Anxiety for Cancer-Related Medical Procedures: A Systematic Narrative Review. J Pain Symptom Manage [Internet]. 2021;61(2):384–94. Available from: https://www.sciencedirect.com/science/article/pii/S0885392420306953
28. Wilson K, Scorsone G. The Use of Virtual Reality Technologies to Reduce Anxiety and

For peer review only

Improve Experience in Chemotherapy Patients During Treatment [Internet]. Vol. 2, Frontiers in Virtual Reality . 2021. Available from: https://www.frontiersin.org/articles/10.3389/frvir.2021.695449

29. Ioannou A, Papastavrou E, Avraamides MN, Charalambous A. Virtual Reality and Symptoms Management of Anxiety, Depression, Fatigue, and Pain: A Systematic Review. SAGE Open Nurs [Internet]. 2020 Jan 1;6:2377960820936163. Available from: https://doi.org/10.1177/2377960820936163

30. Ong TL, Ruppert MM, Akbar M, Rashidi P, Ozrazgat-Baslanti T, Bihorac A, et al. Improving the Intensive Care Patient Experience With Virtual Reality-A Feasibility Study. Crit care Explor. 2020 Jun;2(6):e0122.

31. Groninger H, Stewart D, Wesley D, Cowgill J, Mete M. Virtual reality for management of cancer pain: Study rationale and design. Contemp Clin Trials Commun [Internet]. 2022;26:100895. Available from: https://www.sciencedirect.com/science/article/pii/S2451865422000126

32. Rutkowski S, Czech O, Wrzeciono A, Kiper P, Szczepańska-Gieracha J, Malicka I. Virtual reality as a chemotherapy support in treatment of anxiety and fatigue in patients with cancer: A systematic review and meta-analysis and future research directions. Complement Ther Med [Internet]. 2021;61:102767. Available from: https://www.sciencedirect.com/science/article/pii/S0965229921001084

33. Blomain E, Capaldi DP, Panjwani N, Skinner L, Loo Jr BW. Development of a Smartphone-Based Virtual Reality Platform for Radiation Oncology Patient Education. Int J Radiat Oncol Biol Phys [Internet]. 2021 Nov 1;111(3):e156. Available from: https://doi.org/10.1016/j.ijrobp.2021.07.621

34. Duan J. Development and Evaluation of a Cost-Effective Virtual Reality Tool for Patient Education Before Radiotherapy Treatment. Int J Radiat Oncol Biol Phys [Internet]. 2021 Nov 1;111(3):e156–7. Available from: https://doi.org/10.1016/j.ijrobp.2021.07.622

35. Yap KY-L, Koh DWH, Lee VSJ, Wong LL. Use of virtual reality in the supportive care management of paediatric patients with cancer. Lancet Child Adolesc Heal [Internet]. 2020;4(12):899–908. Available from: https://www.sciencedirect.com/science/article/pii/S2352464220302406

36. Chin C-H, Tseng L-M, Chao T-C, Wang T-J, Wu S-F, Liang S-Y. Self-care as a mediator

For peer review only

between symptom-management self-efficacy and quality of life in women with breast cancer. PLoS One [Internet]. 2021 Feb 4;16(2):e0246430. Available from: https://doi.org/10.1371/journal.pone.0246430

37. Chan A-W, Tetzlaff JM, Gøtzsche PC, Altman DG, Mann H, Berlin JA, et al. SPIRIT 2013 explanation and elaboration: guidance for protocols of clinical trials. BMJ Br Med J [Internet]. 2013 Jan 9;346:e7586. Available from: http://www.bmj.com/content/346/bmj.e7586.abstract

38. Calvert M, King M, Mercieca-Bebber R, Aiyegbusi O, Kyte D, Slade A, et al. SPIRIT-PRO Extension explanation and elaboration: guidelines for inclusion of patient-reported outcomes in protocols of clinical trials. BMJ Open [Internet]. 2021 Jun 1;11(6):e045105. Available from: http://bmjopen.bmj.com/content/11/6/e045105.abstract

39. Boutron I, Altman DG, Moher D, Schulz KF, Ravaud P. CONSORT Statement for Randomized Trials of Nonpharmacologic Treatments: A 2017 Update and a CONSORT Extension for Nonpharmacologic Trial Abstracts. Ann Intern Med [Internet]. 2017 Jun 20;167(1):40–7. Available from: https://www.acpjournals.org/doi/abs/10.7326/M17-0046

40. Faul F, Erdfelder E, Buchner A, Lang A-G. Statistical power analyses using G*Power 3.1: Tests for correlation and regression analyses. Behav Res Methods [Internet]. 2009;41(4):1149–60. Available from: https://doi.org/10.3758/BRM.41.4.1149

41. Artanti ER, Nurjannah I, Subroto S. Validity and reliability of shortened general Comfort questionnaire in indonesian version. Belitung Nurs J [Internet]. 2018 Jun 29;4(4 SE-Original Research Article):366–72. Available from: https://www.belitungraya.org/BRP/index.php/bnj/article/view/437

42. Mumtaz DF, Effendy C, Haryani H. Impact of Pre-Chemotherapy Education with Audio Visual Methods on the Self-Efficacy of Symptom Management in Patients with Cancer. J Cancer Educ [Internet]. 2022;37(5):1546–52. Available from: https://doi.org/10.1007/s13187-021-02006-1

43. Davey HM, Barratt AL, Butow PN, Deeks JJ. A one-item question with a Likert or Visual Analog Scale adequately measured current anxiety. J Clin Epidemiol [Internet]. 2007;60(4):356–60. Available from: https://www.sciencedirect.com/science/article/pii/S0895435606003878

44. Facco E, Zanette G, Favero L, Bacci C, Sivolella S, Cavallin F, et al. Toward the Validation

For peer review only

of Visual Analogue Scale for Anxiety. Anesth Prog [Internet]. 2011 Jan 1;58(1):8–13. Available from: https://doi.org/10.2344/0003-3006-58.1.8

45. Paice JA, Cohen FL. Validity of a verbally administered numeric rating scale to measure cancer pain intensity. Cancer Nurs [Internet]. 1997;20(2). Available from: https://journals.lww.com/cancernursingonline/Fulltext/1997/04000/Validity_of_a_verbally_administered_numeric_rating.2.aspx
46. Brunelli C, Zecca E, Martini C, Campa T, Fagnoni E, Bagnasco M, Lanata L, Caraceni A. Comparison of numerical and verbal rating scales to measure pain exacerbations in patients with chronic cancer pain. Health and quality of life outcomes. 2010 Dec;8:1-8. Available from: https://doi.org/10.1007/s13187-021-02006-1
47. American Heart Association | To be a relentless force for a world of longer, healthier lives [Internet]. www.heart.org. [cited 2022 Dec 09]. Available from: https://www.heart.org/en/
48. Ahmad M, Bani Mohammad E, Anshasi HA. Virtual Reality Technology for Pain and Anxiety Management among Patients with Cancer: A Systematic Review. Pain Manag Nurs [Internet]. 2020;21(6):601–7. Available from: https://www.sciencedirect.com/science/article/pii/S1524904220301168
49. Magill N, Knight R, McCrone P, Ismail K, Landau S. A scoping review of the problems and solutions associated with contamination in trials of complex interventions in mental health. BMC Med Res Methodol [Internet]. 2019;19(1):4. Available from: https://doi.org/10.1186/s12874-018-0646-z
50. Fabi A, Fotia L, Giuseppini F, Gaeta A, Falcicchio C, Giuliani G, et al. The immersive experience of virtual reality during chemotherapy in patients with early breast and ovarian cancers: the Patient's Dream study. Front Oncol. 2022;4271.
51. McNamee R. Regression modelling and other methods to control confounding. Occup Environ Med [Internet]. 2005 Jul 1;62(7):500 LP – 506. Available from: http://oem.bmj.com/content/62/7/500.abstract
52. Pourhoseingholi MA, Baghestani AR, Vahedi M. How to control confounding effects by statistical analysis. Gastroenterology and hepatology from bed to bench. 2012;5(2):79.

For peer review only

Table and Figure Legends

Figure 1. Modified CONSORT Flow Diagram

Figure 2. 360° videos used in the S-VR intervention

Figure 3. Guided imagery leaflet

Table 1. Study outcomes

Table 2. Timeline of data collection and measurements

For peer review only

Table 1. Study outcomes

Outcome	Measurement tool	Score	Interpretation
**Primary**			
Comfort	the Shortened General Comfort Questionnaire, Indonesian version (SGCQ) (41)	Likert scale—1 (strongly disagree) to 6 (strongly agree)	Higher scores indicate higher levels of comfort.
**Secondary**			
Symptom management self-efficacy	the Self-Efficacy of Cancer Symptom Self-Management Scale (SE-CSSMS) (42)	Likert scale—1 (not at all sure) to 10 (very sure)	Higher scores indicate higher levels of self-efficacy.
Anxiety	the Visual Analog Scale for Anxiety (VAS-A) (43,44)	A horizontal line with a scale of 100 mm	Higher scores indicate greater anxiety.
Pain	the Numerical Rating Pain Scale (NPS) (45)	Numerical scale—0 to 10 (11-point scale)	Higher scores indicate greater pain.
Pulse rate	Digital sphygmomanometer	Beats per minute (bpm)	An adult's regular resting heart rate ranges from 60 to 100 bpm.
Blood pressure	Digital sphygmomanometer	Millimeter of mercury (mmHg)	Systolic pressure above 90 mmHg and lower than 120 mmHg and diastolic pressure between 60 mmHg and 80 mmHg

For peer review only

Table 2. Timeline of data collection and measurements

Assessment/measurement instrument	Study period				
	T0	T1	T2	T3	T4
Demographic variables	**X**				
Clinical characteristics	**X**				
SGCQ	**X**				**X**
SEQSM	**X**				**X**
VAS-A	**X**	**X**	**X**	**X**	**X**
NPS	**X**	**X**	**X**	**X**	**X**
Blood pressure	**X**	**X**	**X**	**X**	**X**
Pulse rate	**X**	**X**	**X**	**X**	**X**

T0: Baseline data collection (after prechemotherapy assessment and obtaining signed informed consent); T1: Before intravenous installation/premedication; T2: Before the start of the intervention; T3: Immediately after the intervention; T4: After completion of one chemotherapy cycle

For peer review only

1

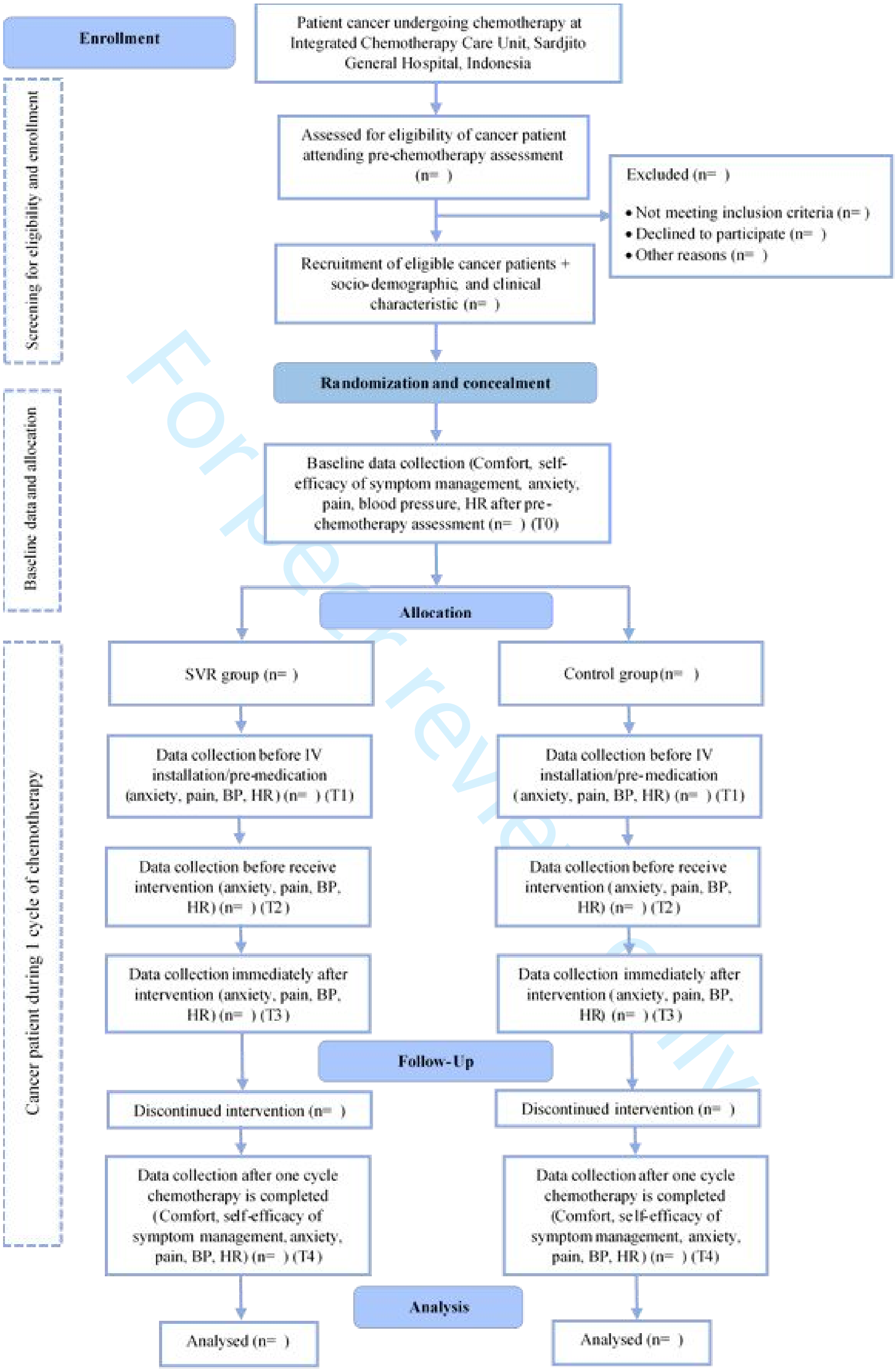

Figure 1. Modified CONSORT Flow Diagram

For peer review only

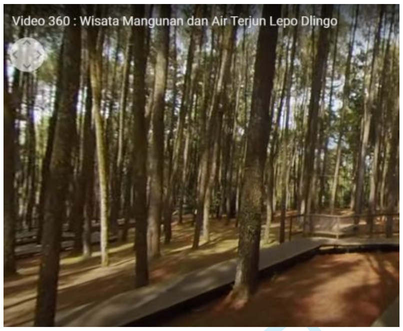

360° Video: Pine Forest and Waterfall in Yogyakarta, Indonesia (https://www.youtube.com/watch?v=OJ5LpBqiRk8)

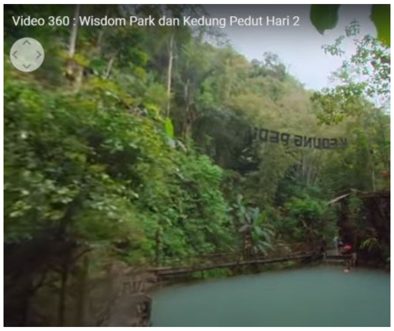

360° Video: Wisdom Park and Natural Pond in Yogyakarta, Indonesia (https://www.youtube.com/watch?v=6A1RTps5fdY)

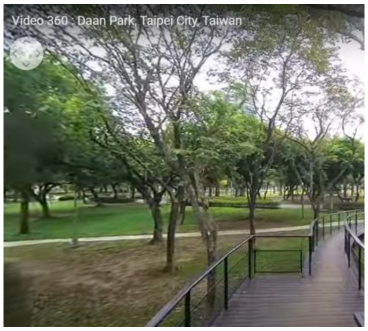

360° Video: Daan Park in Taipei City, Taiwan (https://www.youtube.com/watch?v=-h8JgPONKws)

Figure 2. 360° videos used in the S-VR intervention

For peer review only

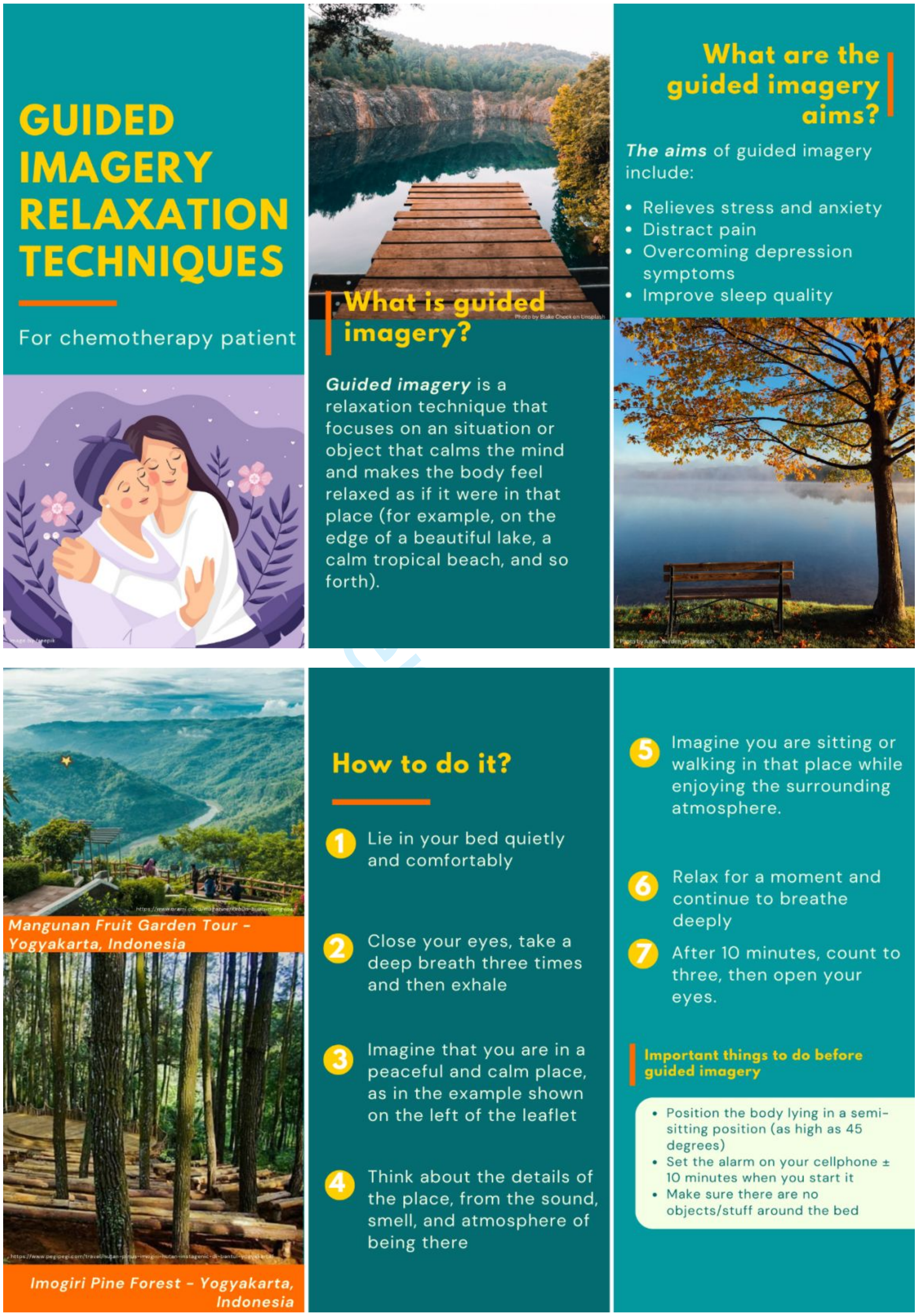

Figure 3. Guided imagery leaflet

CONSORT 2010 checklist of information to include when reporting a randomised trial*

Efficacy of Smartphone-Based Virtual Reality Relaxation in Providing Comfort to Patients with Cancer Undergoing Chemotherapy: Protocol for a Randomized Controlled Trial

Section/Topic	Item No	Checklist item	Reported on page No
**Title and abstract**			
	1a	Identification as a randomised trial in the title	1
	1b	Structured summary of trial design, methods, results, and conclusions (for specific guidance see CONSORT for abstracts)	4 - 5
**Introduction**			
Background and objectives	2a	Scientific background and explanation of rationale	6 – 8
	2b	Specific objectives or hypotheses	8
**Methods**			
Trial design	3a	Description of trial design (such as parallel, factorial) including allocation ratio	9
	3b	Important changes to methods after trial commencement (such as eligibility criteria), with reasons	10 – 11
Participants	4a	Eligibility criteria for participants	10 – 11
	4b	Settings and locations where the data were collected	9
Interventions	5	The interventions for each group with sufficient details to allow replication, including how and when they were actually administered	14 – 15
Outcomes	6a	Completely defined pre-specified primary and secondary outcome measures, including how and when they were assessed	11 – 13
	6b	Any changes to trial outcomes after the trial commenced, with reasons	NA
Sample size	7a	How sample size was determined	10 – 11
	7b	When applicable, explanation of any interim analyses and stopping guidelines	NA
Randomisation:			
Sequence generation	8a	Method used to generate the random allocation sequence	13 – 14
	8b	Type of randomisation; details of any restriction (such as blocking and block size)	13 – 14
Allocation concealment mechanism	9	Mechanism used to implement the random allocation sequence (such as sequentially numbered containers), describing any steps taken to conceal the sequence until interventions were assigned	13 – 14
Implementation	10	Who generated the random allocation sequence, who enrolled participants, and who assigned participants to	13 – 14

For peer review only

For peer review only - http://bmjopen.bmj.com/site/about/guidelines.xhtml

		interventions	
Blinding	11a	If done, who was blinded after assignment to interventions (for example, participants, care providers, those assessing outcomes) and how	13 – 14
	11b	If relevant, description of the similarity of interventions	15
Statistical methods	12a	Statistical methods used to compare groups for primary and secondary outcomes	16 – 17
	12b	Methods for additional analyses, such as subgroup analyses and adjusted analyses	17
**Results**			
Participant flow (a diagram is strongly recommended)	13a	For each group, the numbers of participants who were randomly assigned, received intended treatment, and were analysed for the primary outcome	Figure 1
	13b	For each group, losses and exclusions after randomisation, together with reasons	NA
Recruitment	14a	Dates defining the periods of recruitment and follow-up	NA
	14b	Why the trial ended or was stopped	NA
Baseline data	15	A table showing baseline demographic and clinical characteristics for each group	NA
Numbers analysed	16	For each group, number of participants (denominator) included in each analysis and whether the analysis was by original assigned groups	NA
Outcomes and estimation	17a	For each primary and secondary outcome, results for each group, and the estimated effect size and its precision (such as 95% confidence interval)	NA
	17b	For binary outcomes, presentation of both absolute and relative effect sizes is recommended	NA
Ancillary analyses	18	Results of any other analyses performed, including subgroup analyses and adjusted analyses, distinguishing pre-specified from exploratory	NA
Harms	19	All important harms or unintended effects in each group (for specific guidance see CONSORT for harms)	NA
**Discussion**			
Limitations	20	Trial limitations, addressing sources of potential bias, imprecision, and, if relevant, multiplicity of analyses	NA
Generalisability	21	Generalisability (external validity, applicability) of the trial findings	NA
Interpretation	22	Interpretation consistent with results, balancing benefits and harms, and considering other relevant evidence	NA
**Other information**			
Registration	23	Registration number and name of trial registry	2 & 18
Protocol	24	Where the full trial protocol can be accessed, if available	18
Funding	25	Sources of funding and other support (such as supply of drugs), role of funders	18 – 19

For peer review only

For peer review only - http://bmjopen.bmj.com/site/about/guidelines.xhtml

*We strongly recommend reading this statement in conjunction with the CONSORT 2010 Explanation and Elaboration for important clarifications on all the items. If relevant, we also recommend reading CONSORT extensions for cluster randomised trials, non-inferiority and equivalence trials, non-pharmacological treatments, herbal interventions, and pragmatic trials. Additional extensions are forthcoming: for those and for up to date references relevant to this checklist, see www.consort-statement.org.

For peer review only

For peer review only - http://bmjopen.bmj.com/site/about/guidelines.xhtml

BMJ Open

Efficacy of Smartphone-Based Virtual Reality Relaxation in Providing Comfort to Patients with Cancer Undergoing Chemotherapy in the Oncology Outpatient Setting: Protocol for a Randomized Controlled Trial

Journal:	*BMJ Open*
Manuscript ID	bmjopen-2023-074506.R1
Article Type:	Protocol
Date Submitted by the Author:	28-Jun-2023
Complete List of Authors:	Gautama, Made Satya Nugraha ; Taipei Medical University, School of Nursing, College of Nursing, ; Universitas Gadjah Mada, Master of Nursing Program, Faculty of Medicine Public Health and Nursing, Haryani, Haryani; Universitas Gadjah Mada, Department of Surgical Medical Nursing, Faculty of Medicine, Public Health and Nursing, Huang, Tsai-Wei ; Taipei Medical University, School of Nursing, College of Nursing; Taipei Municipal Wan-Fang Hospital, Research Center in Nursing Clinical Practice, Department of Nursing
**Primary Subject Heading**:	Nursing
Secondary Subject Heading:	Evidence based practice
Keywords:	Virtual Reality, CHEMOTHERAPY, Nursing Care, Adult palliative care < PALLIATIVE CARE

SCHOLARONE™
Manuscripts

Efficacy of Smartphone-Based Virtual Reality Relaxation in Providing Comfort to Patients with Cancer Undergoing Chemotherapy in the Oncology Outpatient Setting: Protocol for a Randomized Controlled Trial

Made Satya Nugraha Gautama,[1,2] Haryani Haryani,[3] Tsai-Wei Huang[1,4,5,6]

[1]School of Nursing, College of Nursing, Taipei Medical University, Taipei, Taiwan

[2]Master of Nursing Program, Faculty of Medicine Public Health and Nursing, Universitas Gadjah Mada, Yogyakarta, Indonesia

[3]Department of Surgical Medical Nursing, Faculty of Medicine, Public Health and Nursing, Universitas Gadjah Mada, Yogyakarta, Indonesia

[4]Research Center in Nursing Clinical Practice, Department of Nursing, Wan Fang Hospital, Taipei Medical University, Taipei, Taiwan

[5]Department of Nursing, Wan Fang Hospital, Taipei Medical University, Taipei, Taiwan

[6]Cochrane Taiwan, Taipei Medical University, Taipei, Taiwan

***Corresponding Author:** Tsai-Wei Huang, RN, Ph.D.
School of Nursing, College of Nursing, Taipei Medical University 250, Wuxing Street, Taipei City, Taiwan, 11031

Tel: +886-2-27361661#6350

Fax: +886-2-3772842

E-mail: tsaiwei@tmu.edu.tw

ORCID: https://orcid.org/0000-0002-6722-1153

For peer review only

Short running head: Smartphone-Based Virtual Reality Relaxation for Comfort in Chemotherapy

Data Availability Statement

All data reported in this manuscript can be found in the literature cited in the text.

Conflicts of Interest

The authors have no conflicts of interest to disclose.

Ethics Approval and Consent to Participate

The study was conducted in accordance with the Declaration of Helsinki (as revised in 2013). Ethics approval was obtained by the Medical and Health Research Ethics Committee (MHREC) of the Faculty of Medicine, Public Health and Nursing of Universitas Gadjah Mada—Dr. Sardjito General Hospital, Yogyakarta, Institutional Review Board (IRB) (Approval number: KE/FK/0301/EC/2023). The protocol was approved at the ClinicalTrial.gov, Registry of Clinical Trials on 2023/05/08 (Identifiers: NCT05756465). Any modification to the protocol will be approved by the ethics committee prior to its implementation. All participants will sign a written informed consent form.

For peer review only

Consent for publication

The participant consent forms will be made available upon request to the corresponding author.

Acknowledgement

This manuscript was edited by Wallace Academic Editing.

For peer review only

Authors' Contributions

SNG, HH, and TWH conceptualized and designed the study. TWH and HH provided methodological advice related to the study design. The manuscript was written by SNG and TWH and reviewed by all authors. All authors designed the statistical analysis. All authors made critical revisions to the manuscript and provided final approval for the manuscript.

Funding

This work was supported by National Science and Technology Council grant number NSTC 111-2622-E-038 -003 for APC support.

Abstract

Introduction: Patients with cancer undergoing chemotherapy experience various physical and psychological problems and discomfort. Virtual reality (VR) can be used in technology-based nonpharmacological therapy that can serve as a potential distractor in the symptom management of patients with cancer undergoing chemotherapy. We propose a Smartphone-Based Virtual Reality Relaxation (S-VR) technique as a complementary modality to provide comfort to patients with cancer, and we will evaluate its effect on patients with cancer undergoing chemotherapy.

Methods and analysis: We will recruit 80 patients from the One Day Chemotherapy "Tulip" Unit of Dr. Sardjito General Hospital, Yogyakarta, Indonesia. This will be a two-arm parallel randomized controlled trial, with a 1:1 allocation and the primary outcome assessor blinded. This study will be divided into two group: (2) an intervention group, with the participants receiving 360° panoramic video content and music relaxation intervention through a VR device (head-mounted display) placed on their head during chemotherapy for ±10 minutes plus standard care, and (2) a control group, with the participants receiving guided imagery relaxation therapy in the form of leaflet plus standard care. We will measure the outcomes after one chemotherapy cycle for each participant. The primary outcome is the effectiveness of the S-VR in improving the comfort of patients. The secondary outcome is the effect of the S-VR

For peer review only

on the patients' symptom management self-efficacy, pain, anxiety, blood pressure (SBP and DBP), and pulse rate.

Ethics and dissemination: This study was approved by the Medical and Health Research Ethics Committee (MHREC) of the Faculty of Medicine, Public Health and Nursing of Universitas Gadjah Mada—Dr. Sardjito General Hospital, Yogyakarta, Institutional Review Board (IRB) (Approval number: KE/FK/0301/EC/2023). Written informed consent will be obtained from all participants who enroll in the study. Dissemination will be conducted through peer-reviewed publications and conference presentations.

Trial Registration Number: NCT05756465

Keywords: Virtual Reality; chemotherapy; relaxation; comfort; self-efficacy

Strengths and Limitations of this Study

- In addition to patient-reported outcomes, the present randomized controlled trial will assess patients' bio-parameters, such as blood pressure and heart rate, as well as cybersickness symptom evaluation due to the VR device.
- We will increase the sample size and make it larger than minimum sample size to increase statistical power.

For peer review only

- Using multivariate statistical analysis, potential confounding variables that may influence the primary outcome will be adjusted for.
- Due to the inherent properties of VR intervention, blinding is inapplicable.
- The principal objective of this study was to enhance patient comfort during the brief duration of chemotherapy within the chemotherapy room.

For peer review only

INTRODUCTION

The cancer incidence in Indonesia (136.2/100,000 population) is the 8th highest in Southeast Asia and 23rd highest in Asia [1]. According to Basic Health Surveillance (Riskesdas) [2], the prevalence of cancer in Indonesia increased from 1.4 in 2013 to 1.49 in 2018. Of the total of 396,914 cancer cases in Indonesia in 2020, the top three most common cancer types were breast cancer (65,858 cases; 16.6%), cervical cancer (36,633 cases; 9.2%), and lung cancer (21,392 cases; 8.8%) [3]. The region with the highest prevalence of cancer is the Special Region of Yogyakarta Province (4.86/1000 population), and 23% of the patients with cancer in this region receive chemotherapy, which is the second-most-common cancer management strategy after surgery [2].

Patients undergoing chemotherapy experience various adverse effects [4]. Moreover, they may experience physical and psychological discomfort caused by the treatment drugs, invasive procedures, and chemotherapy environments. In general, 86% of patients with cancer experience pain, nausea, vomiting, mucositis, diarrhea, and alopecia [5,6]. Other side effects include psychological problems, such as anxiety, feelings of hopelessness, depression, anger, fear, vulnerability, and a sense of loss of control [7]. Management of the side effects of chemotherapy by using drugs is not fully effective and may cause other side effects,[8] which can cause further patient discomfort [8,9].

For peer review only

Comfort is defined as a lack of pain, distress, worry, and uneasiness, and it is one of the most crucial factors in holistic nursing care [10,11]. Focusing on patients' biophysiological aspects (e.g., symptom management) as well as psychospiritual, environmental, and sociocultural aspects related to healing is crucial for maintaining their well-being [12]. Kolcaba explained that in addition to providing basic care to patients, nurses must provide special and unique nursing care to each patient to increase patient comfort [13,14]. Patients with cancer require physical, social, and spiritual support and a comfortable environment to have a sense of life meaning [12,15,16]. Meeting the comfort needs of patients with cancer positively affects the patients' satisfaction with the services they receive, their treatment adherence, and their overall quality of life [11,17].

Many nonpharmacological or alternative approaches are used in the management of chemotherapy side effects[18]. Some studies have reported that nonpharmacological therapies, such as music therapy, acupuncture, massage, guided imagery, hypnosis, acupressure, and virtual reality (VR) therapy, can alleviate physical and psychological symptoms in patients with cancer [19,20]. However, most nonpharmacological therapies involve the use of conventional methods; few have incorporated technologies, such as VR, into alternative therapy [21]. The use of VR in both cancer research and therapy remains limited [22].

For peer review only

Virtual Reality (VR) therapy is gaining popularity in alternative medicine due to its ability to overcome certain limitations of traditional therapy [23,24]. Firstly, VR offers an immersive and interactive environment that fosters a sense of presence and transports individuals to a different reality, potentially reducing anxiety and pain perception [25]. Research has shown that this immersive experience can create a therapeutic effect. Secondly, VR therapy provides a customizable and controlled environment that can be tailored to meet the specific needs of individual patients [26]. This personalized approach allows for a more targeted and effective treatment experience. By adapting the virtual scenarios and activities to the patient's preferences and requirements, VR therapy offers a unique and individualized intervention. Although VR has been partially applied in cancer care, especially to reduce patient anxiety and pain during invasive procedures [21,27].

Although scientific evidence of the effectiveness of using VR in the treatment of cancer continues to grow, findings regarding the use of smartphone-integrated VR remain limited [28,29]. Therefore, in the present study, we proposed an innovative strategy for the management of discomfort in patients undergoing chemotherapy by using smartphone-based VR relaxation (S-VR) with content including natural panoramic views and three-dimensional relaxation music. It is anticipated that this alternative therapy will help alleviate symptoms in chemotherapy patients by reducing

For peer review only

anxiety and diverting attention from pain. However, further research is needed to explore the full potential and efficacy of smartphone-based VR therapy in managing chemotherapy-related symptoms.

The primary objective of this study is to determine the efficacy of S-VR in increasing comfort in patients with cancer undergoing chemotherapy. The secondary objectives of this study are to assess the effects of S-VR on the symptom management self-efficacy, anxiety, pain, and vital signs (pulse and blood pressure) of patients with cancer undergoing chemotherapy. We hypothesize that relative to the control group, the patient group that receives the S-VR intervention will experience greater improvements in their comfort levels.

For peer review only

METHODS AND ANALYSIS

Design

This study is a two-arm parallel randomized controlled trial with a 1:1 allocation. The study will divide the patients into two groups: the intervention group, in which the patients will receive S-VR therapy through a VR device (head-mounted display) mounted on the patients' heads during chemotherapy plus standard care (daily routine care in administration of chemotherapy drugs), and the control group, in which the patients will receive standard care and guided imagery therapy with leaflets containing instructions for techniques for relaxing through image visualization. The group allocation will be implemented using the envelope concealment method, and the outcome assessor will be blinded to the group allocation during data collection. The protocol has been designed in accordance with the Standard Protocol Items: Recommendations for Interventional Trials and its checklist [30,31]. Figure 1 presents the modified CONSORT (2017) flow diagram [32].

Setting

The Special Region of Yogyakarta (DIY) Province will be the study location because it has the highest prevalence of cancer in Indonesia. The national prevalence of cancer is 1.79/1000 population, whereas the prevalence in the DIY Province is 4.86/1000

For peer review only

population, with 23% of these patients undergoing chemotherapy treatment [2]. This study will be conducted at the One Day Chemotherapy Polyclinic “Tulip” Integrated Cancer Unit of Dr. Sardjito General Hospital, Yogyakarta, Indonesia. According to the annual report of the service performance of the Integrated Cancer Unit of Dr. Sardjito General Hospital, the number of visits by patients with cancer was 100,614 people in 2019, an increase of 19.8% from the previous year.

Patient and public involvement

The protocol has been designed in close collaboration between the School of Nursing, Taipei Medical University, and the Master of Nursing Programme, Universitas Gadjah Mada, with strong support from Dr. Sardjito General Hospital, Yogyakarta. We work with cancer patients undergoing chemotherapy under the Integrated Cancer Unit at Dr. Sardjito General Hospital Yogyakarta, Indonesia. A patient with cancer undergoing chemotherapy was enrolled in this intervention programme and completed the study evaluation. Valuable input, preference, and evaluation on the part of both patients and hospital institutions have been invaluable in the development of this intervention, thus, we hope that the findings from this RCT will be disseminated to local, regional, and national policymakers as well as an international scientific audience.

Population and Sample

For peer review only

The target population is patients with cancer who will undergo chemotherapy at the "Tulip" Integrated Cancer Unit of Dr. Sardjito General Hospital. The inclusion criteria for this study are as follows:

1. Being a patient with cancer receiving chemotherapy in all cycles.
2. Being aged ≥18 years.
3. Having a performance status (ECOG Score) of 0–2.
4. Being able to understand and sign the informed consent form.

Respondents who meet the following exclusion criteria will be excluded from the study:

1. Being a patient with cancer with a history of skull structure or cervical spine abnormalities that may complicate the use of VR devices.
2. Being a cancer patient with cognitive, visual, and hearing impairment; or having a chemo port.
3. Being a patient with a history of epilepsy, seizures, vertigo, or visually induced motion sickness.

Sample Size Calculation

The sample size was calculated using free-to use software, namely G*Power for Windows Version 3.1.9.6 [33]. The effect size is 0.81, type 1 error (α) = 5% (two-tailed), power (1-β) 90%, and the allocation ratio between the groups is 1:1. The

For peer review only - http://bmjopen.bmj.com/site/about/guidelines.xhtml

effect size was set with reference to previous studies with similar outcomes [9]. According to the G*Power calculation, the study sample size should be 34 per group. With consideration of an expected dropout rate of 15%, the sample size was increased to 40 per group.

The participant dropout criteria are as follows:

1. The patient being unwilling to continue the sequence of the study process (lost to follow-up).
2. The patients experiencing drug extravasation incidents or severe side effects during chemotherapy.

The consecutive sampling technique will be applied. This nonprobability sampling method involves recruiting all individuals from the accessible population during a given time interval or for a specific sample size. Patients with cancer who have scheduled chemotherapy and meet the requirements of the data collection time will be asked to participate in the study.

Participant Characteristics and Study Outcomes

Participant characteristics

A questionnaire will be used to collect patient demographic data, including age, sex, education level, marital status, occupation, income, and assistance during chemotherapy, and clinical characteristics, including the type of cancer, body surface

For peer review only

area (height and weight), cancer stage, duration of diagnosis, chemotherapy cycle, and ECOG score.

Outcomes

The primary and secondary outcomes are summarized in Table 1.

Comfort of cancer patients is measured using the shortened general comfort questionnaire (SGCQ) instrument by Chatarine Kolcaba in 2006 which has been tested for validity and reliability in Indonesian version by Artanti et al. [34]. The validity test reported that Content validity index (CVI) of item (I-CVI) and scale (S-CVI) score were both 1 (valid). Cronbach's alpha score showed 0.769, meaning that the SGCQ Indonesian version is reliable. The SGCQ instrument describes individual comfort by assigning a Likert score to 28 items. Likert scores range from 1 (strongly disagree) to 6 (strongly agree). The highest total score is 168, and the lowest is 28.

Self – efficacy is measured by using the Self-Efficacy of Cancer Symptom Self-Management Scale (SE – CSSMS),[35] which consists of 6 question items with a value range of 1 (not at all sure) to 10 (very sure). SE - CSSMS has been tested for validity and reliability on cancer patients in Indonesia [35]. The content validity index (CVI) is 0.94. The r-value is 0.467, which is greater than the r-table value of 0.12, so these results state that the six items in the symptom management self-efficacy

For peer review only

instrument are valid. The reliability test results showed that the Cronbach alpha value is 0.90 (> 0.70), so the measuring instrument is reliable.

Anxiety is measured using the Visual Analog Scale for Anxiety (VAS – A) in the form of a horizontal line with a scale of 100 millimeter or 10 centimeters, with the left by the words “not at all anxious” and on the right by “extremely anxious” [36,37]. VAS-A has a sensitivity of 76.8%, specifications of 64.9%, and internal reliability consistency (Cronbach Alpha ≥0.7 [36,37].

Pain is measured using the numeric rating scale (NRS). The NRS consist of numerical scale; from 0 to 100 (101 – point scale), 0 to 10 (11 – point scale) or 1 to 10 (10 – point scale). This study will use a 0 – 10 NRS due to it provides more sufficient sensitivity to measure pain in most cancer patients.[38] The NRS has been declared valid and reliable for subjective measurement of cancer pain, both due to adverse effects of cytotoxic agents, cancer treatment procedures (surgery, radiotherapy, and chemotherapy), and the malignancy of the cancer itself [39].

Pulse rate and blood pressure (systolic and diastolic pressure) are measured using an automatic digital sphygmomanometer. According to American Heart Association, the regular resting heart rate for adults is 60 to 100 beats per minute, and systolic blood pressure is above 90 mm Hg and less than 120 mm Hg, with a diastolic pressure between 60 mm Hg and less than 80 mm Hg [40].

For peer review only

Comfort and self-efficacy for symptom management will be measured at two time points: at baseline (before chemotherapy premedication) and posttest (after the chemotherapy session is completed). Anxiety, pain, and vital signs (pulse rate and blood pressure) will be measured at five time points: thrice at pretest (baseline data collection, before the installation of intravenous access, and before administration of VR intervention) and twice at posttest (immediately after intervention and after the chemotherapy session is completed). The timeline of the data collection and measurement is presented in Table 2.

Randomization and Concealment

The researchers will screen to identify patients who are eligible to participate in the study. The patients' medical records will be evaluated to verify their baseline information (e.g., anthropometric data, laboratory data, patient identification data, and laboratory results). An allocation sequence will be generated through simple randomization of the study participants by using a formula on the Microsoft Excel program (the RANDBETWEEN function), and the allocation sequence will be concealed in sealed envelopes. Thereafter, the participants in the intervention and control groups will receive envelopes containing random numbers generated using the randomization procedure. The outcome assessor (the nurse appointed by the

For peer review only

researcher to be responsible for outcome data collection) and data analysts will be blinded to the participant allocation. Due to the technical nature of the VR interventions; it was difficult to blind the study participants. Thus, to minimize potential methodological bias, the outcome assessor and data analysts will be blinded [41].

Patient Recruitment

Recruitment will be conducted from April to July 2023. Patients who are scheduled for chemotherapy will be assessed by the nurse on duty, who will review their latest laboratory results, medical records, vital signs, and electrocardiogram results, to determine whether they are eligible to undergo chemotherapy. The patients who pass the screening process will be asked whether they are willing to be involved in the study and will be asked to sign the informed consent form. Patients who are willing and who sign the informed consent form will be given a sealed envelope containing a random number indicating their group allocation. The participants will be evaluated (pretest) by the outcome assessor (research nurse) before they enter the chemotherapy room. After the evaluation, the researcher will open each participant's envelope and see the random number that they have been assigned (without the knowledge of the participants or the outcome assessor). If the number assigned is between 1 and 40, the participant will be directed to the chemotherapy room on the east side of the hospital

For peer review only

(intervention group); if the number is between 41 and 80, the participant will be directed to the chemotherapy room on the west side of the hospital (control group). We will for the participants in the intervention and control groups to be directed to different chemotherapy rooms, and a research assistant (RA) will accompany the participants to the rooms to minimize contamination of information between participants, between participants and intervention providers and between the nurses or doctors on duty [42].

Intervention (S-VR + standard care)

Participants in the intervention arm will receive immersive VR intervention with a VR box (Shinecon 6.0 VR Box Virtual Reality Glasses with headphones). S-VR is a virtual relaxation and distraction therapy administered through a smartphone-based VR device (head-mounted display) mounted to the head of the patient with cancer during chemotherapy. The intervention involves a virtual environment in which 360° videos of natural panoramas with traditional and classical music (noncopyright) are provided. The S-VR device is paired with a smartphone with either an Android (minimum generation 11, Android 4.4 KitKat) or iOS (version 12, minimum screen size of 5.5 inch and maximum of 6.0-inch, screen resolution of at least 1080 × 1920 pixels) operating system and the ability to connect to WIFI or the Internet and with the YouTube app installed, which they will use to access the 360° videos. The S-VR

For peer review only

content comprises original 360° videos approximately 10 minutes long and produced by the researchers by using video editing software. The video duration was determined on the basis of a previous study by Fabi et al.[43] in which this duration was demonstrated to be effective in preventing patient saturation related to content and preventing motion sickness. The 360° videos present panoramic views of Yogyakarta, Indonesia, and Taipei, Taiwan (Figure 2). The RA will provide information about the S-VR intervention for approximately 5 minutes, assist in attaching the S-VR devices to the patient, and conduct the intervention for approximately 10 minutes.

Control (Leaflet + standard care)

In the control group, participants will be provided with standard care and guided imagery leaflets, which will provide information on the meaning, benefits, and methods of completing guided imagery relaxation therapy during chemotherapy. The RA will guide the participants through the practices of the guided imagery relaxation therapy described in the leaflet for ±10 minutes (Figure 3).

Compensation

Compensation will be provided by the researchers to all respondents after the chemotherapy session in the form of a package containing food, beverage, and a small

For peer review only

towel. These objects are placed in a small bag along with a note of appreciation for participation in this research.

Safety and Side Effect Management

The intervention of this study is not associated with any significant side effects because it does not involve invasive procedures and is administered for a short period (1 chemotherapy cycle). However, we predict that the participants will feel discomfort both while using the VR and during chemotherapy and will record their levels of discomfort on a monitoring sheet. The Virtual Reality Symptom Questionnaire (VRSQ), which was developed by Ames in 2005 [44], will be administered to patients after the VR intervention in order to assess the possibility of cybersickness, a form of motion sickness caused by exposure to VR. The questionnaire evaluates eight general physical side effects (general discomfort, fatigue, boredom, drowsiness, headache, dizziness, concentration difficulties, and nausea) and five visual effects (tired eyes, aching eyes, eyestrain, blurred vision, and difficulties focusing). The scoring was modified to a four-point scale (the original score consisted of a six-point scale), from 0 to 4 (represented of none, mild, moderate, severe,) with 0 indicating the absence of the symptom and 4 indicating its severity. During the study, the researchers will clearly indicate that they can be contacted should the participants have any problems or complaints or if something unexpected occurs.

For peer review only

Data Analysis

The collected data will be processed using IBM SPSS statistical software version 23 for Windows through editing, coding, and data entry processes. The statistician will be blinded to the study outcomes and will conduct analyses on the basis of the labeling of the groups. For the treatment group, the demographic and clinical variables recorded at enrollment will be tabulated. The descriptive statistics will be presented as numbers, means, standard deviations, medians, minimums, and maximums. Homogeneity and normality tests will be conducted before the bivariate analysis test. A homogeneity test for categorical data (age, sex, education level, marital status, occupation, income, the presence of a family member or caregiver during chemotherapy, type of cancer, stage of cancer, duration of cancer diagnosis, chemotherapy cycle, and ECOG scores) will be performed using the chi-square or Fisher's test, whereas a homogeneity test for numerical data (height and weight) will be performed using Levene's test if the data are normally distributed and using the Mann–Whitney U test if the data are not normally distributed. Both groups will be considered to be homogeneous if $p > 0.05$. Bivariate analysis will be used to determine the differences in the mean scores for comfort level and symptom management self-efficacy before and after treatment in the intervention and control groups. If the data distribution is normal, we will use a paired *t* test and independent *t*

For peer review only

test. However, if the data distribution is not normal, the nonparametric Wilcoxon and Mann–Whitney U tests will be used if significant differences are identified ($p < 0.05$). For anxiety, pain, and vital-sign (blood pressure and pulse rate) outcome measurement analysis, General Linear Model-Repeated Measure (GLM-RM) statistics will be used. The GLM-RM is a statistical test used for the comparative analysis of two unpaired numerical data groups with more than two measurements and is applied when the effect of a treatment or intervention is measured at different time points. If the data are not normally distributed, the repeated Mann–Whitney alternative test with Bonferroni correction will be used. The present study will use statistical method to adjust for potentially confounding effects of the S-VR intervention on the patient's outcomes. These confounders include age, gender, education, cancer stage, chemotherapy cycle, assistance during chemotherapy, and comorbidities. Multivariate model can handle identified confounders and minimize the bias effect of the intervention [45]. Linear regression analysis will be applied by using the enter method. Adjustment by using this analysis can clarify that how much the confounders in the model distort the intervention and outcome [46].

Ethics and Dissemination

The present study was approved by the Medical and Health Research Ethics Committee (MHREC) Faculty of Medicine, Public Health and Nursing Universitas

For peer review only

Gadjah Mada—Dr. Sardjito General Hospital, Yogyakarta, IRB (Approval number: KE/FK/0301/EC/2023). All participants enrolled in the study will be asked to provide written informed consent, which will include a statement requesting their willingness to allow the data to be share. All information related to the identity of the participants will be kept confidential by the researchers. No posttrial care has been planned. The study results will be published in a peer-reviewed scientific journal. The results will not indicate the identity of any participant. Authorship eligibility was based on an individual making substantial contributions to the study and manuscript review. Access to the final data set will be granted to the primary investigator and coinvestigators. The study protocol is available on clinicaltrials.gov (ClinicalTrials.gov Identifier: NCT05756465). The study is scheduled to start recruitment at Dr. Sardjito General Hospital in Indonesia in April 2023. After the implementation of the intervention, data collection, and statistical analysis, we expect to submit the results to a peer-reviewed journal in October 2023.

For peer review only

Contributors

SNG developed the initial trial concepts and drafted the manuscript. TWH and HH contributed to the development of the manuscript. All authors have critically revised the manuscript for essential intellectual content. All authors have read and approved the submitted version of the manuscript.

Funding

This work was supported by National Science and Technology Council grant number NSTC 111-2622-E-038 -003 for APC support.

Competing Interests

None to declare.

Patient Consent for Publication

Not required.

Provenance and Peer Review

Not commissioned; external peer review

For peer review only

REFERENCES

1 Ministry of Health Republic of Indonesia. 2019.https://www.kemkes.go.id/article/view/19020100003/hari-kanker-sedunia-2019.html

2 Basic Health Survailance/Riset Kesehatan Dasar tahun 2018. 2018. http://repository.bkpk.kemkes.go.id/3514/1/Laporan Riskesdas 2018 Nasional.pdf

3 Globocan. New Global Cancer Data: Indonesia. 2020. https://gco.iarc.fr/today/data/factsheets/populations/360-indonesia-fact-sheets.pdf

4 Thiagarajan M, Chan CMH, Fuang HG, *et al.* Symptom prevalence and related distress in cancer patients undergoing chemotherapy. *Asian Pacific Journal of Cancer Prevention* 2016;**17**:171–6.

5 Pearce A, Haas M, Viney R, *et al.* Incidence and severity of self-reported chemotherapy side effects in routine care: A prospective cohort study. *PLoS One* 2017;**12**:e0184360–e0184360.https://doi.org/10.1371/journal.pone.0184360

For peer review only

6 Şahin ZA, Ergüney S. Effect on Symptom Management Education Receiving Patients of Chemotherapy. *Journal of Cancer Education* 2016;**31**:101–7. doi:10.1007/s13187-015-0801-8

7 Society AC. Chemotherapy side effects | american cancer society. 2023.https://www.cancer.org/treatment/treatments-and-side-effects/treatment-types/chemotherapy/chemotherapy-side-effects.html

8 Keim-Malpass J, Levine B, Danhauer SC, *et al.* Work-related perceptions and quality of life among breast cancer survivors. *Psychooncology* 2016;**25**:873–6. doi:10.1002/pon.3951

9 Bilgiç Ş, Acaroğlu R. Effects of Listening to Music on the Comfort of Chemotherapy Patients. *West J Nurs Res* 2016;**39**:745–62. doi:10.1177/0193945916660527

10 Kolcaba KY. Holistic comfort: Operationalizing the construct as a nurse-sensitive outcome. *Advances in nursing science* 1992;**15**:1–10.https://www.researchgate.net/profile/Katharine-Kolcaba/publication/21635629_Holistic_comfort_operationalizing_the_construct_as_a_nurse-sensitive_outcome/links/54eb45810cf2082851bd7130/Holistic-comfort-operationalizing-the-construct-as-a-nurse-sensitive-ou

For peer review only

11 Can A, Hintistan S. An Assessment of the Comfort Level of Cancer Patients with Receiving Chemotheraphy. *Bezmialem Science* 2021;**9**:310–6. doi:10.14235/bas.galenos.2020.4226

12 Smith MC, Parker ME. *Nursing Theories and Nursing Practice 4th ed.* Philadelphia, PA: : F.A. Davis 2015.

13 Kolcaba KY. The Art of Comfort Care. *Image: the Journal of Nursing Scholarship* 1995;**27**:287–9. doi:https://doi.org/10.1111/j.1547-5069.1995.tb00889.x

14 Nuraini T, Gayatri D, Rachmawati İ. Comfort assessment of cancer patient in palliative care: A nursing perspective. *Int J Caring Sci* 2017;**10**:209.https://internationaljournalofcaringsciences.org/docs/24_tuti_original_10_1.pdf

15 Kolcaba K. *Comfort Theory and Practice: A Vision for Holistic Health Care and Research*. New York, NY: : Springer Publishing Company 2003.

16 Cheng F, Wang W. Factors influencing comfort level in head and neck neoplasm patients receiving radiotherapy. *Int J Nurs Sci* 2014;**1**:394–9. doi:https://doi.org/10.1016/j.ijnss.2014.10.009

17 Miao J, Liu M, Ma J, *et al.* Effectiveness of Comfort Nursing Combined with Continuous Nursing on Patients with Colorectal Cancer Chemotherapy.

For peer review only

Evidence-Based Complementary and Alternative Medicine 2022;**2022**:9647325. doi:10.1155/2022/9647325

18 Prigerson HG, Bao Y, Shah MA, *et al.* Chemotherapy Use, Performance Status, and Quality of Life at the End of Life. *JAMA Oncol* 2015;**1**:778–84. doi:10.1001/jamaoncol.2015.2378

19 Bani Mohammad E, Ahmad M. Virtual reality as a distraction technique for pain and anxiety among patients with breast cancer: A randomized control trial. *Palliat Support Care* 2019;**17**:29–34. doi:DOI: 10.1017/S1478951518000639

20 Scates D, Dickinson JI, Sullivan K, *et al.* Using Nature-Inspired Virtual Reality as a Distraction to Reduce Stress and Pain Among Cancer Patients. *Environ Behav* 2020;**52**:895–918. doi:10.1177/0013916520916259

21 Chirico A, Lucidi F, De Laurentiis M, *et al.* Virtual Reality in Health System: Beyond Entertainment. A Mini-Review on the Efficacy of VR During Cancer Treatment. *J Cell Physiol* 2016;**231**:275–87. doi:https://doi.org/10.1002/jcp.25117

22 Chirico A, Maiorano P, Indovina P, *et al.* Virtual reality and music therapy as distraction interventions to alleviate anxiety and improve mood states in breast cancer patients during chemotherapy. *J Cell Physiol* 2020;**235**:5353–62. doi:https://doi.org/10.1002/jcp.29422

For peer review only

23 Stetz MAJ, Ries RI, Folen RA. Virtual reality supporting psychological health. In: *Advanced Computational Intelligence Paradigms in Healthcare 6. Virtual Reality in Psychotherapy, Rehabilitation, and Assessment*. Springer 2011. 13–29.

24 Morina N, Ijntema H, Meyerbröker K, *et al.* Can virtual reality exposure therapy gains be generalized to real-life? A meta-analysis of studies applying behavioral assessments. *Behaviour Research and Therapy* 2015;**74**:18–24. doi:https://doi.org/10.1016/j.brat.2015.08.010

25 Rubio-Tamayo JL, Barrio MG, García FG. Immersive Environments and Virtual Reality: Systematic Review and Advances in Communication, Interaction and Simulation. *Multimodal Technologies and Interaction 2017, Vol 1, Page 21* 2017;**1**:21. doi:10.3390/MTI1040021

26 Boeldt D, McMahon E, McFaul M, *et al.* Using Virtual Reality Exposure Therapy to Enhance Treatment of Anxiety Disorders: Identifying Areas of Clinical Adoption and Potential Obstacles. *Front Psychiatry* 2019;**10**:476694. doi:10.3389/FPSYT.2019.00773/BIBTEX

27 Huang Q, Lin J, Han R, *et al.* Using Virtual Reality Exposure Therapy in Pain Management: A Systematic Review and Meta-Analysis of Randomized

For peer review only

Controlled Trials. *Value in Health* 2022;**25**:288–301. doi:https://doi.org/10.1016/j.jval.2021.04.1285

28 Groninger H, Stewart D, Wesley D, *et al.* Virtual reality for management of cancer pain: Study rationale and design. *Contemp Clin Trials Commun* 2022;**26**:100895. doi:https://doi.org/10.1016/j.conctc.2022.100895

29 Rutkowski S, Czech O, Wrzeciono A, *et al.* Virtual reality as a chemotherapy support in treatment of anxiety and fatigue in patients with cancer: A systematic review and meta-analysis and future research directions. *Complement Ther Med* 2021;**61**:102767. doi:https://doi.org/10.1016/j.ctim.2021.102767

30 Chan A-W, Tetzlaff JM, Gøtzsche PC, *et al.* SPIRIT 2013 explanation and elaboration: guidance for protocols of clinical trials. *BMJ : British Medical Journal* 2013;**346**:e7586–e7586. doi:10.1136/bmj.e7586

31 Calvert M, King M, Mercieca-Bebber R, *et al.* SPIRIT-PRO Extension explanation and elaboration: guidelines for inclusion of patient-reported outcomes in protocols of clinical trials. *BMJ Open* 2021;**11**:e045105–e045105. doi:10.1136/bmjopen-2020-045105

32 Boutron I, Altman DG, Moher D, *et al.* CONSORT Statement for Randomized Trials of Nonpharmacologic Treatments: A 2017 Update and a CONSORT

For peer review only

Extension for Nonpharmacologic Trial Abstracts. *Ann Intern Med* 2017;**167**:40–7. doi:10.7326/M17-0046

33 Faul F, Erdfelder E, Buchner A, *et al.* Statistical power analyses using G*Power 3.1: Tests for correlation and regression analyses. *Behav Res Methods* 2009;**41**:1149–60. doi:10.3758/BRM.41.4.1149

34 Artanti ER, Nurjannah I, Subroto S. Validity and reliability of shortened general Comfort questionnaire in indonesian version. *Belitung Nurs J* 2018;**4**:366–72. doi:10.33546/bnj.437

35 Mumtaz DF, Effendy C, Haryani H. Impact of Pre-Chemotherapy Education with Audio Visual Methods on the Self-Efficacy of Symptom Management in Patients with Cancer. *Journal of Cancer Education* 2022;**37**:1546–52. doi:10.1007/s13187-021-02006-1

36 Davey HM, Barratt AL, Butow PN, *et al.* A one-item question with a Likert or Visual Analog Scale adequately measured current anxiety. *J Clin Epidemiol* 2007;**60**:356–60. doi:https://doi.org/10.1016/j.jclinepi.2006.07.015

37 Facco E, Zanette G, Favero L, *et al.* Toward the Validation of Visual Analogue Scale for Anxiety. *Anesth Prog* 2011;**58**:8–13. doi:10.2344/0003-3006-58.1.8

38 Paice JA, Cohen FL. Validity of a verbally administered numeric rating scale to measure cancer pain intensity. *Cancer Nurs*

For peer review only

1997;**20**.https://journals.lww.com/cancernursingonline/Fulltext/1997/04000/Validity_of_a_verbally_administered_numeric_rating.2.aspx

39 Brunelli C, Zecca E, Martini C, *et al.* Comparison of numerical and verbal rating scales to measure pain exacerbations in patients with chronic cancer pain. *Health Qual Life Outcomes* 2010;**8**. doi:10.1186/1477-7525-8-42

40 American Heart Association | To be a relentless force for a world of longer, healthier lives. https://www.heart.org/en/ (accessed 17 Jun 2023).

41 Ahmad M, Bani Mohammad E, Anshasi HA. Virtual Reality Technology for Pain and Anxiety Management among Patients with Cancer: A Systematic Review. *Pain Management Nursing* 2020;**21**:601–7. doi:https://doi.org/10.1016/j.pmn.2020.04.002

42 Magill N, Knight R, McCrone P, *et al.* A scoping review of the problems and solutions associated with contamination in trials of complex interventions in mental health. *BMC Med Res Methodol* 2019;**19**:4. doi:10.1186/s12874-018-0646-z

43 Fabi A, Fotia L, Giuseppini F, *et al.* The immersive experience of virtual reality during chemotherapy in patients with early breast and ovarian cancers: the Patient's Dream study. *Front Oncol* 2022;:4271.

For peer review only

44 Ames SL, Wolffsohn JS, Mcbrien NA. The development of a symptom questionnaire for assessing virtual reality viewing using a head-mounted display. *Optom Vis Sci* 2005;**82**:168–76. doi:10.1097/01.OPX.0000156307.95086.6

45 McNamee R. Regression modelling and other methods to control confounding. *Occup Environ Med* 2005;**62**:500 LP – 506. doi:10.1136/oem.2002.001115

46 Pourhoseingholi MA, Baghestani AR, Vahedi M. How to control confounding effects by statistical analysis. *Gastroenterol Hepatol Bed Bench* 2012;**5**:79./pmc/articles/PMC4017459/ (accessed 17 Jun 2023).

For peer review only

Table and Figure Legends

Figure 1. Modified CONSORT Flow Diagram

Figure 2. 360° videos used in the S-VR intervention

Figure 3. Guided imagery leaflet

Table 1. Study outcomes

Table 2. Timeline of data collection and measurements

For peer review only

Table 1. Study outcomes

Outcome	Measurement tool	Score	Interpretation
**Primary**			
Comfort	the Shortened General Comfort Questionnaire, Indonesian version (SGCQ) (41)	Likert scale—1 (strongly disagree) to 6 (strongly agree)	Higher scores indicate higher levels of comfort.
**Secondary**			
Symptom management self-efficacy	the Self-Efficacy of Cancer Symptom Self-Management Scale (SE-CSSMS) (42)	Likert scale—1 (not at all sure) to 10 (very sure)	Higher scores indicate higher levels of self-efficacy.
Anxiety	the Visual Analog Scale for Anxiety (VAS-A) (43,44)	A horizontal line with a scale of 100 mm	Higher scores indicate greater anxiety.
Pain	the Numerical Rating Pain Scale (NPS) (45)	Numerical scale—0 to 10 (11-point scale)	Higher scores indicate greater pain.
Pulse rate	Digital sphygmomanometer	Beats per minute (bpm)	An adult's regular resting heart rate ranges from 60 to 100 bpm.
Blood pressure	Digital sphygmomanometer	Millimeter of mercury (mmHg)	Systolic pressure above 90 mmHg and lower than 120 mmHg

For peer review only

	and diastolic pressure between 60 mmHg and 80 mmHg

For peer review only

Table 2. Timeline of data collection and measurements

Assessment/measurement instrument	Study period				
	T0	T1	T2	T3	T4
Demographic variables	**X**				
Clinical characteristics	**X**				
SGCQ	**X**				**X**
SEQSM	**X**				**X**
VAS-A	**X**	**X**	**X**	**X**	**X**
NPS	**X**	**X**	**X**	**X**	**X**
Blood pressure	**X**	**X**	**X**	**X**	**X**
Pulse rate	**X**	**X**	**X**	**X**	**X**

T0: Baseline data collection (after prechemotherapy assessment and obtaining signed informed consent); T1: Before intravenous installation/premedication; T2: Before the start of the intervention; T3: Immediately after the intervention; T4: After completion of one chemotherapy cycle

For peer review only

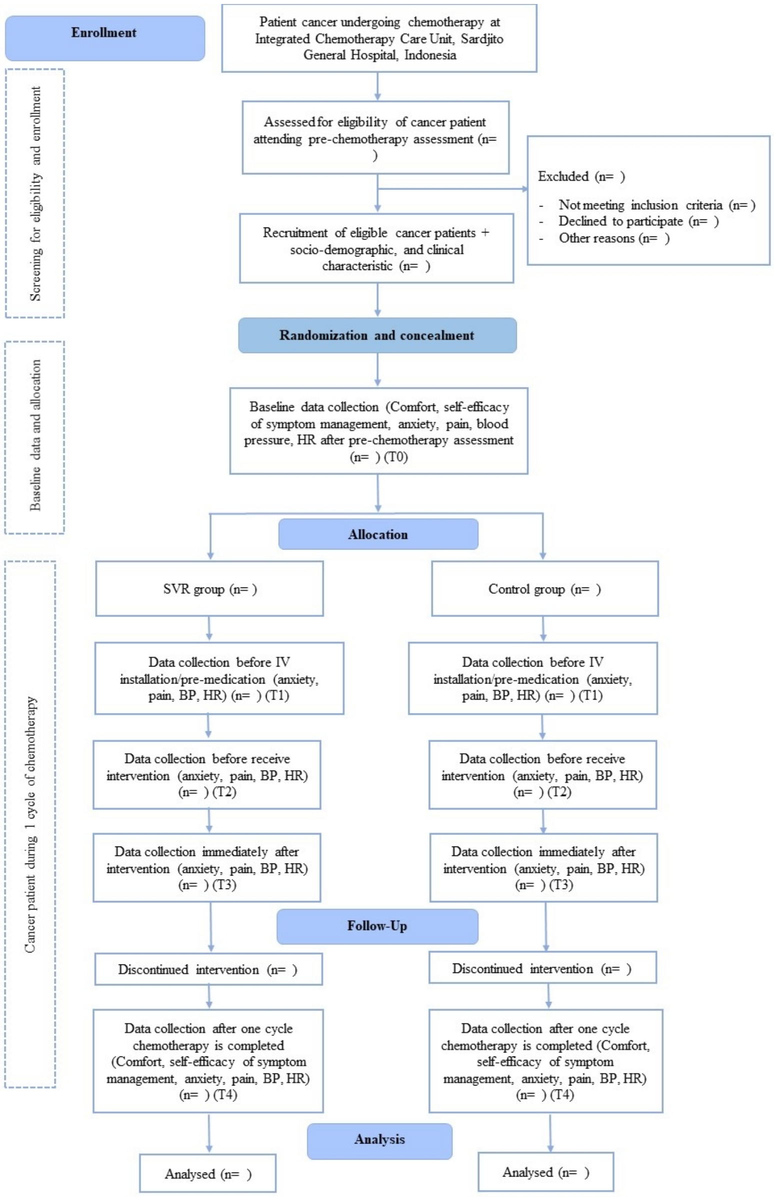

Figure 1. Modified CONSORT Flow Diagram

85x113mm (300 x 300 DPI)

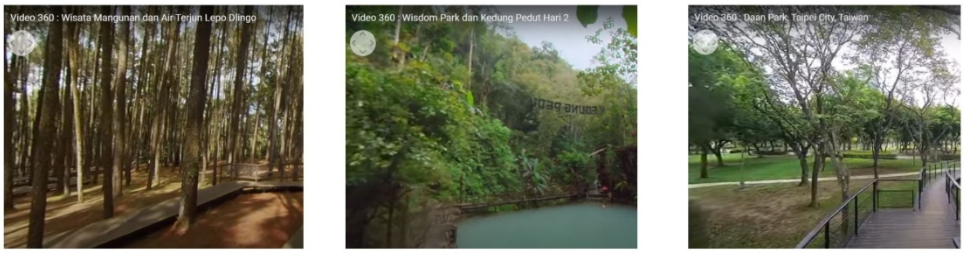

Figure 2. 360° videos used in the S-VR intervention

174x87mm (300 x 300 DPI)

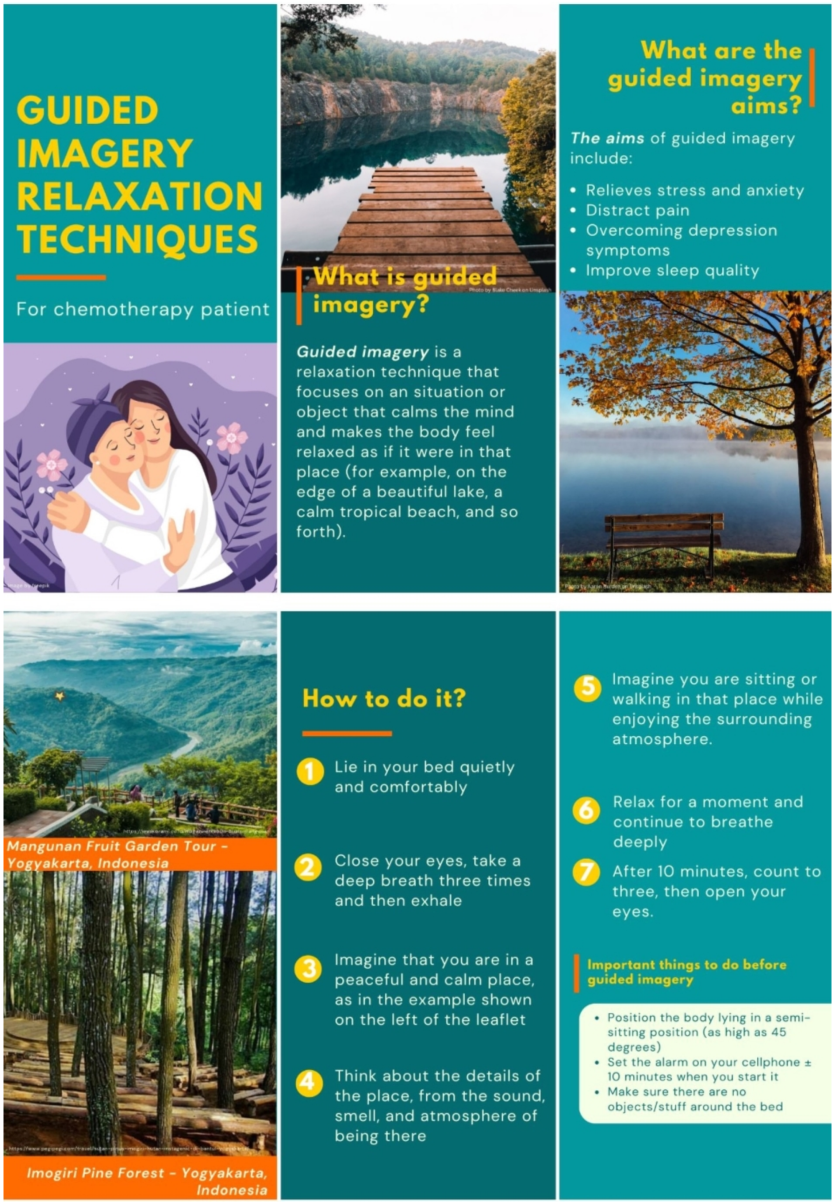

Figure 3. Guided imagery leaflet

133x192mm (300 x 300 DPI)

CONSORT 2010 checklist of information to include when reporting a randomised trial*

Efficacy of Smartphone-Based Virtual Reality Relaxation in Providing Comfort to Patients with Cancer Undergoing Chemotherapy: Protocol for a Randomized Controlled Trial

Section/Topic	Item No	Checklist item	Reported on page No
**Title and abstract**			
	1a	Identification as a randomised trial in the title	1
	1b	Structured summary of trial design, methods, results, and conclusions (for specific guidance see CONSORT for abstracts)	4 - 5
**Introduction**			
Background and objectives	2a	Scientific background and explanation of rationale	6 – 8
	2b	Specific objectives or hypotheses	8
**Methods**			
Trial design	3a	Description of trial design (such as parallel, factorial) including allocation ratio	9
	3b	Important changes to methods after trial commencement (such as eligibility criteria), with reasons	10 – 11
Participants	4a	Eligibility criteria for participants	10 – 11
	4b	Settings and locations where the data were collected	9
Interventions	5	The interventions for each group with sufficient details to allow replication, including how and when they were actually administered	14 – 15
Outcomes	6a	Completely defined pre-specified primary and secondary outcome measures, including how and when they were assessed	11 – 13
	6b	Any changes to trial outcomes after the trial commenced, with reasons	NA
Sample size	7a	How sample size was determined	10 – 11
	7b	When applicable, explanation of any interim analyses and stopping guidelines	NA
Randomisation:			
Sequence generation	8a	Method used to generate the random allocation sequence	13 – 14
	8b	Type of randomisation; details of any restriction (such as blocking and block size)	13 – 14
Allocation concealment mechanism	9	Mechanism used to implement the random allocation sequence (such as sequentially numbered containers), describing any steps taken to conceal the sequence until interventions were assigned	13 – 14
Implementation	10	Who generated the random allocation sequence, who enrolled participants, and who assigned participants to	13 – 14

For peer review only - http://bmjopen.bmj.com/site/about/guidelines.xhtml

		interventions	
Blinding	11a	If done, who was blinded after assignment to interventions (for example, participants, care providers, those assessing outcomes) and how	13 – 14
	11b	If relevant, description of the similarity of interventions	15
Statistical methods	12a	Statistical methods used to compare groups for primary and secondary outcomes	16 – 17
	12b	Methods for additional analyses, such as subgroup analyses and adjusted analyses	17
**Results**			
Participant flow (a diagram is strongly recommended)	13a	For each group, the numbers of participants who were randomly assigned, received intended treatment, and were analysed for the primary outcome	Figure 1
	13b	For each group, losses and exclusions after randomisation, together with reasons	NA
Recruitment	14a	Dates defining the periods of recruitment and follow-up	NA
	14b	Why the trial ended or was stopped	NA
Baseline data	15	A table showing baseline demographic and clinical characteristics for each group	NA
Numbers analysed	16	For each group, number of participants (denominator) included in each analysis and whether the analysis was by original assigned groups	NA
Outcomes and estimation	17a	For each primary and secondary outcome, results for each group, and the estimated effect size and its precision (such as 95% confidence interval)	NA
	17b	For binary outcomes, presentation of both absolute and relative effect sizes is recommended	NA
Ancillary analyses	18	Results of any other analyses performed, including subgroup analyses and adjusted analyses, distinguishing pre-specified from exploratory	NA
Harms	19	All important harms or unintended effects in each group (for specific guidance see CONSORT for harms)	NA
**Discussion**			
Limitations	20	Trial limitations, addressing sources of potential bias, imprecision, and, if relevant, multiplicity of analyses	NA
Generalisability	21	Generalisability (external validity, applicability) of the trial findings	NA
Interpretation	22	Interpretation consistent with results, balancing benefits and harms, and considering other relevant evidence	NA
**Other information**			
Registration	23	Registration number and name of trial registry	2 & 18
Protocol	24	Where the full trial protocol can be accessed, if available	18
Funding	25	Sources of funding and other support (such as supply of drugs), role of funders	18 – 19

For peer review only - http://bmjopen.bmj.com/site/about/guidelines.xhtml

*We strongly recommend reading this statement in conjunction with the CONSORT 2010 Explanation and Elaboration for important clarifications on all the items. If relevant, we also recommend reading CONSORT extensions for cluster randomised trials, non-inferiority and equivalence trials, non-pharmacological treatments, herbal interventions, and pragmatic trials. Additional extensions are forthcoming: for those and for up to date references relevant to this checklist, see www.consort-statement.org.

For peer review only

SPIRIT
STANDARD PROTOCOL ITEMS: RECOMMENDATIONS FOR INTERVENTIONAL TRIALS

SPIRIT 2013 Checklist: Recommended items to address in a clinical trial protocol and related documents*

Section/item	Item No	Description	Pages
**Administrative information**			
Title	1	Descriptive title identifying the study design, population, interventions, and, if applicable, trial acronym	1
Trial registration	2a	Trial identifier and registry name. If not yet registered, name of intended registry	4 – 5
	2b	All items from the World Health Organization Trial Registration Data Set	NA
Protocol version	3	Date and version identifier	2
Funding	4	Sources and types of financial, material, and other support	3
Roles and responsibilities	5a	Names, affiliations, and roles of protocol contributors	3
	5b	Name and contact information for the trial sponsor	NA
	5c	Role of study sponsor and funders, if any, in study design; collection, management, analysis, and interpretation of data; writing of the report; and the decision to submit the report for publication, including whether they will have ultimate authority over any of these activities	NA
	5d	Composition, roles, and responsibilities of the coordinating centre, steering committee, endpoint adjudication committee, data management team, and other individuals or groups overseeing the trial, if applicable (see Item 21a for data monitoring committee)	NA
**Introduction**			
Background and rationale	6a	Description of research question and justification for undertaking the trial, including summary of relevant studies (published and unpublished) examining benefits and harms for each intervention	8 – 9
	6b	Explanation for choice of comparators	8 – 9
Objectives	7	Specific objectives or hypotheses	10

For peer review only

Trial design	8	Description of trial design including type of trial (eg, parallel group, crossover, factorial, single group), allocation ratio, and framework (eg, superiority, equivalence, noninferiority, exploratory)	10
**Methods: Participants, interventions, and outcomes**			
Study setting	9	Description of study settings (eg, community clinic, academic hospital) and list of countries where data will be collected. Reference to where list of study sites can be obtained	11
Eligibility criteria	10	Inclusion and exclusion criteria for participants. If applicable, eligibility criteria for study centres and individuals who will perform the interventions (eg, surgeons, psychotherapists)	13 – 14
Interventions	11a	Interventions for each group with sufficient detail to allow replication, including how and when they will be administered	19 – 20
	11b	Criteria for discontinuing or modifying allocated interventions for a given trial participant (eg, drug dose change in response to harms, participant request, or improving/worsening disease)	13
	11c	Strategies to improve adherence to intervention protocols, and any procedures for monitoring adherence (eg, drug tablet return, laboratory tests)	NA
	11d	Relevant concomitant care and interventions that are permitted or prohibited during the trial	NA
Outcomes	12	Primary, secondary, and other outcomes, including the specific measurement variable (eg, systolic blood pressure), analysis metric (eg, change from baseline, final value, time to event), method of aggregation (eg, median, proportion), and time point for each outcome. Explanation of the clinical relevance of chosen efficacy and harm outcomes is strongly recommended	15 – 17
Participant timeline	13	Time schedule of enrolment, interventions (including any run-ins and washouts), assessments, and visits for participants. A schematic diagram is highly recommended (see Figure)	Table 2
Sample size	14	Estimated number of participants needed to achieve study objectives and how it was determined, including clinical and statistical assumptions supporting any sample size calculations	13-14

For peer review only - http://bmjopen.bmj.com/site/about/guidelines.xhtml

Recruitment	15	Strategies for achieving adequate participant enrolment to reach target sample size	13-14
**Methods: Assignment of interventions (for controlled trials)**			
Allocation:			
Sequence generation	16a	Method of generating the allocation sequence (eg, computer-generated random numbers), and list of any factors for stratification. To reduce predictability of a random sequence, details of any planned restriction (eg, blocking) should be provided in a separate document that is unavailable to those who enrol participants or assign interventions	20
Allocation concealment mechanism	16b	Mechanism of implementing the allocation sequence (eg, central telephone; sequentially numbered, opaque, sealed envelopes), describing any steps to conceal the sequence until interventions are assigned	17
Implementation	16c	Who will generate the allocation sequence, who will enrol participants, and who will assign participants to interventions	17-18
Blinding (masking)	17a	Who will be blinded after assignment to interventions (eg, trial participants, care providers, outcome assessors, data analysts), and how	17-18
	17b	If blinded, circumstances under which unblinding is permissible, and procedure for revealing a participant's allocated intervention during the trial	17-18
**Methods: Data collection, management, and analysis**			
Data collection methods	18a	Plans for assessment and collection of outcome, baseline, and other trial data, including any related processes to promote data quality (eg, duplicate measurements, training of assessors) and a description of study instruments (eg, questionnaires, laboratory tests) along with their reliability and validity, if known. Reference to where data collection forms can be found, if not in the protocol	12-17
	18b	Plans to promote participant retention and complete follow-up, including list of any outcome data to be collected for participants who discontinue or deviate from intervention protocols	NA
Data management	19	Plans for data entry, coding, security, and storage, including any related processes to promote data quality (eg, double data entry; range checks for data values). Reference to where details of data management procedures can be found, if not in the protocol	NA

For peer review only - http://bmjopen.bmj.com/site/about/guidelines.xhtml

Statistical methods	20a	Statistical methods for analysing primary and secondary outcomes. Reference to where other details of the statistical analysis plan can be found, if not in the protocol	22-23
	20b	Methods for any additional analyses (eg, subgroup and adjusted analyses)	22-23
	20c	Definition of analysis population relating to protocol non-adherence (eg, as randomised analysis), and any statistical methods to handle missing data (eg, multiple imputation)	NA
**Methods: Monitoring**			
Data monitoring	21a	Composition of data monitoring committee (DMC); summary of its role and reporting structure; statement of whether it is independent from the sponsor and competing interests; and reference to where further details about its charter can be found, if not in the protocol. Alternatively, an explanation of why a DMC is not needed	NA
	21b	Description of any interim analyses and stopping guidelines, including who will have access to these interim results and make the final decision to terminate the trial	NA
Harms	22	Plans for collecting, assessing, reporting, and managing solicited and spontaneously reported adverse events and other unintended effects of trial interventions or trial conduct	21
Auditing	23	Frequency and procedures for auditing trial conduct, if any, and whether the process will be independent from investigators and the sponsor	NA
**Ethics and dissemination**			
Research ethics approval	24	Plans for seeking research ethics committee/institutional review board (REC/IRB) approval	23-24
Protocol amendments	25	Plans for communicating important protocol modifications (eg, changes to eligibility criteria, outcomes, analyses) to relevant parties (eg, investigators, REC/IRBs, trial participants, trial registries, journals, regulators)	NA
Consent or assent	26a	Who will obtain informed consent or assent from potential trial participants or authorised surrogates, and how (see Item 32)	13
	26b	Additional consent provisions for collection and use of participant data and biological specimens in ancillary studies, if applicable	NA

only

For peer review only - http://bmjopen.bmj.com/site/about/guidelines.xhtml

Confidentiality	27	How personal information about potential and enrolled participants will be collected, shared, and maintained in order to protect confidentiality before, during, and after the trial	22-24
Declaration of interests	28	Financial and other competing interests for principal investigators for the overall trial and each study site	25
Access to data	29	Statement of who will have access to the final trial dataset, and disclosure of contractual agreements that limit such access for investigators	NA
Ancillary and post-trial care	30	Provisions, if any, for ancillary and post-trial care, and for compensation to those who suffer harm from trial participation	NA
Dissemination policy	31a	Plans for investigators and sponsor to communicate trial results to participants, healthcare professionals, the public, and other relevant groups (eg, via publication, reporting in results databases, or other data sharing arrangements), including any publication restrictions	23-24
	31b	Authorship eligibility guidelines and any intended use of professional writers	NA
	31c	Plans, if any, for granting public access to the full protocol, participant-level dataset, and statistical code	NA
**Appendices**			
Informed consent materials	32	Model consent form and other related documentation given to participants and authorised surrogates	NA
Biological specimens	33	Plans for collection, laboratory evaluation, and storage of biological specimens for genetic or molecular analysis in the current trial and for future use in ancillary studies, if applicable	NA

*It is strongly recommended that this checklist be read in conjunction with the SPIRIT 2013 Explanation & Elaboration for important clarification on the items. Amendments to the protocol should be tracked and dated. The SPIRIT checklist is copyrighted by the SPIRIT Group under the Creative Commons "Attribution-NonCommercial-NoDerivs 3.0 Unported" license.

For peer review only - http://bmjopen.bmj.com/site/about/guidelines.xhtml

BMJ Open

Efficacy of Smartphone-Based Virtual Reality Relaxation in Providing Comfort to Cancer Patients Undergoing Chemotherapy in Oncology Outpatient Setting in Indonesia: Protocol for a Randomized Controlled Trial

Journal:	*BMJ Open*
Manuscript ID	bmjopen-2023-074506.R2
Article Type:	Protocol
Date Submitted by the Author:	12-Jul-2023
Complete List of Authors:	Gautama, Made Satya Nugraha ; Taipei Medical University, School of Nursing, College of Nursing, ; Universitas Gadjah Mada, Master of Nursing Program, Faculty of Medicine Public Health and Nursing, Haryani, Haryani; Universitas Gadjah Mada, Department of Surgical Medical Nursing, Faculty of Medicine, Public Health and Nursing, Huang, Tsai-Wei ; Taipei Medical University, School of Nursing, College of Nursing; Taipei Municipal Wan-Fang Hospital, Research Center in Nursing Clinical Practice, Department of Nursing
**Primary Subject Heading**:	Nursing
Secondary Subject Heading:	Evidence based practice
Keywords:	Virtual Reality, CHEMOTHERAPY, Nursing Care, Adult palliative care < PALLIATIVE CARE

SCHOLARONE™
Manuscripts

Efficacy of Smartphone-Based Virtual Reality Relaxation in Providing Comfort to Cancer Patients Undergoing Chemotherapy in Oncology Outpatient Setting in Indonesia: Protocol for a Randomized Controlled Trial

Made Satya Nugraha Gautama,[1,2] Haryani Haryani,[3] Tsai-Wei Huang[1,4,5,6]

[1]School of Nursing, College of Nursing, Taipei Medical University, Taipei, Taiwan

[2]Master of Nursing Program, Faculty of Medicine Public Health and Nursing, Universitas Gadjah Mada, Yogyakarta, Indonesia

[3]Department of Surgical Medical Nursing, Faculty of Medicine, Public Health and Nursing, Universitas Gadjah Mada, Yogyakarta, Indonesia

[4]Research Center in Nursing Clinical Practice, Department of Nursing, Wan Fang Hospital, Taipei Medical University, Taipei, Taiwan

[5]Department of Nursing, Wan Fang Hospital, Taipei Medical University, Taipei, Taiwan

[6]Cochrane Taiwan, Taipei Medical University, Taipei, Taiwan

***Corresponding Author:** Tsai-Wei Huang, RN, Ph.D.
School of Nursing, College of Nursing, Taipei Medical University 250, Wuxing Street, Taipei City, Taiwan, 11031

Tel: +886-2-27361661#6350

Fax: +886-2-3772842

E-mail: tsaiwei@tmu.edu.tw

ORCID: https://orcid.org/0000-0002-6722-1153

For peer review only

Short running head: Smartphone-Based Virtual Reality Relaxation for Comfort in Chemotherapy

Data Availability Statement

All data reported in this manuscript can be found in the literature cited in the text.

Conflicts of Interest

The authors have no conflicts of interest to disclose.

Ethics Approval and Consent to Participate

The study was conducted in accordance with the Declaration of Helsinki (as revised in 2013). Ethics approval was obtained by the Medical and Health Research Ethics Committee (MHREC) of the Faculty of Medicine, Public Health and Nursing of Universitas Gadjah Mada—Dr. Sardjito General Hospital, Yogyakarta, Institutional Review Board (IRB) (Approval number: KE/FK/0301/EC/2023). The protocol was approved at the ClinicalTrial.gov, Registry of Clinical Trials on 2023/05/08 (Identifiers: NCT05756465). Any modification to the protocol will be approved by the ethics committee prior to its implementation. All participants will sign a written informed consent form.

For peer review only

Consent for publication

The participant consent forms will be made available upon request to the corresponding author.

Acknowledgement

This manuscript was edited by Wallace Academic Editing.

Authors' Contributions

SNG, HH, and TWH conceptualized and designed the study. TWH and HH provided methodological advice related to the study design. The manuscript was written by SNG and TWH and reviewed by all authors. All authors designed the statistical analysis. All authors made critical revisions to the manuscript and provided final approval for the manuscript.

Funding

This work was supported by the National Science and Technology Council in Taiwan grant number NSTC 111-2622-E-038 -003 for APC support.

For peer review only

Abstract

Introduction: Patients with cancer undergoing chemotherapy experience various physical and psychological problems and discomfort. Virtual reality (VR) can be used in technology-based nonpharmacological therapy that can serve as a potential distractor in the symptom management of patients with cancer undergoing chemotherapy. We propose a Smartphone-Based Virtual Reality Relaxation (S-VR) technique as a complementary modality to provide comfort to patients with cancer, and we will evaluate its effect on patients with cancer undergoing chemotherapy.

Methods and analysis: We will recruit 80 patients from the One Day Chemotherapy "Tulip" Center of Dr. Sardjito General Hospital, Yogyakarta, Indonesia. This will be a two-arm parallel randomized controlled trial, with a 1:1 allocation and the primary outcome assessor blinded. This study will be divided into two groups: (2) an intervention group, with participants receiving 360 ° panoramic video content and music relaxation intervention through a VR device (head-mounted display) placed on their head during chemotherapy for ±10 minutes plus standard care, and (2) a control group, with participants receiving guided imagery relaxation therapy in the form of a leaflet plus standard care. We will measure the outcomes after one chemotherapy cycle for each participant. The primary outcome is the effectiveness of the S-VR in improving the comfort of patients. The secondary outcome is the effect of the S-VR

For peer review only

on the patients' symptom management self-efficacy, pain, anxiety, blood pressure (SBP and DBP), and pulse rate.

Ethics and dissemination: This study was approved by the Medical and Health Research Ethics Committee (MHREC) of the Faculty of Medicine, Public Health and Nursing of Universitas Gadjah Mada—Dr. Sardjito General Hospital, Yogyakarta, Institutional Review Board (IRB) (Approval number: KE/FK/0301/EC/2023). Written informed consent will be obtained from all participants who enroll in the study. Dissemination will be conducted through peer-reviewed publications and conference presentations.

Trial Registration Number: NCT05756465

Keywords: Virtual Reality; chemotherapy; relaxation; comfort; self-efficacy

Strengths and Limitations of this Study

- In addition to patient-reported outcomes, the present randomized controlled trial will assess patients' bio-parameters, such as blood pressure and heart rate, as well as cybersickness symptom evaluation due to the VR device.
- We will increase the sample size and make it larger than minimum sample size to increase statistical power.

For peer review only

- Using multivariate statistical analysis, potential confounding variables that may influence the primary outcome will be adjusted for.
- Due to the inherent properties of VR intervention, blinding is inapplicable.
- The principal objective of this study was to enhance patient comfort during the brief duration of chemotherapy within the chemotherapy room.
- We will not assess the patients' pre-existing psychological conditions, which may affect their response to Virtual Reality (VR) intervention.
- This study conducts in a single institution, which may limit the generalizability of the findings to other settings and populations.

For peer review only

INTRODUCTION

The cancer incidence in Indonesia (136.2/100,000 population) is the 8th highest in Southeast Asia and 23rd highest in Asia [1]. According to Basic Health Surveillance (Riskesdas) [2], the prevalence of cancer in Indonesia increased from 1.4 in 2013 to 1.49 in 2018. Of the total of 396,914 cancer cases in Indonesia in 2020, the top three most common cancer types were breast cancer (65,858 cases; 16.6%), cervical cancer (36,633 cases; 9.2%), and lung cancer (21,392 cases; 8.8%) [3]. The region with the highest prevalence of cancer is the Special Region of Yogyakarta Province (4.86/1000 population), and 23% of the patients with cancer in this region receive chemotherapy, which is the second-most-common cancer management strategy after surgery [2].

Patients undergoing chemotherapy experience various adverse effects [4]. Moreover, they may experience physical and psychological discomfort caused by the treatment drugs, invasive procedures, and chemotherapy environments. In general, 86% of patients with cancer experience pain, nausea, vomiting, mucositis, diarrhea, and alopecia [5,6]. Other side effects include psychological problems, such as anxiety, feelings of hopelessness, depression, anger, fear, vulnerability, and a sense of loss of control [7]. Management of the side effects of chemotherapy by using drugs is not fully effective and may cause other side effects,[8] which can cause further patient discomfort [8,9].

For peer review only

Comfort is defined as a lack of pain, distress, worry, and uneasiness, and it is one of the most crucial factors in holistic nursing care [10,11]. Focusing on patients' biophysiological aspects (e.g., symptom management) as well as psychospiritual, environmental, and sociocultural aspects related to healing is crucial for maintaining their well-being [12]. Kolcaba explained that in addition to providing basic care to patients, nurses must provide special and unique nursing care to each patient to increase patient comfort [13,14]. Patients with cancer require physical, social, and spiritual support and a comfortable environment to have a sense of life meaning [12,15,16]. Meeting the comfort needs of patients with cancer positively affects the patients' satisfaction with the services they receive, their treatment adherence, and their overall quality of life [11,17].

Many nonpharmacological or alternative approaches are used in the management of chemotherapy side effects[18]. Some studies have reported that nonpharmacological therapies, such as music therapy, acupuncture, massage, guided imagery, hypnosis, acupressure, and virtual reality (VR) therapy, can alleviate physical and psychological symptoms in patients with cancer [19,20]. However, most nonpharmacological therapies involve the use of conventional methods; few have incorporated technologies, such as VR, into alternative therapy [21]. The use of VR in both cancer research and therapy remains limited [22].

For peer review only

Virtual Reality (VR) therapy is gaining popularity in alternative medicine due to its ability to overcome certain limitations of traditional therapy [23,24]. Firstly, VR offers an immersive and interactive environment that fosters a sense of presence and transports individuals to a different reality, potentially reducing anxiety and pain perception [25]. Research has shown that this immersive experience can create a therapeutic effect. Secondly, VR therapy provides a customizable and controlled environment that can be tailored to meet the specific needs of individual patients [26]. This personalized approach allows for a more targeted and effective treatment experience. By adapting the virtual scenarios and activities to the patient's preferences and requirements, VR therapy offers a unique and individualized intervention. Although VR has been partially applied in cancer care, especially to reduce patient anxiety and pain during invasive procedures [21,27].

Although scientific evidence of the effectiveness of using VR in the treatment of cancer continues to grow, findings regarding the use of smartphone-integrated VR remain limited [28,29]. Therefore, in the present study, we proposed an innovative strategy for the management of discomfort in patients undergoing chemotherapy by using smartphone-based VR relaxation (S-VR) with content including natural panoramic views and three-dimensional relaxation music. It is anticipated that this alternative therapy will help alleviate symptoms in chemotherapy patients by reducing

For peer review only

anxiety and diverting attention from pain. However, further research is needed to explore the full potential and efficacy of smartphone-based VR therapy in managing chemotherapy-related symptoms.

The primary objective of this study is to determine the efficacy of S-VR in increasing comfort in patients with cancer undergoing chemotherapy. The secondary objectives of this study are to assess the effects of S-VR on the symptom management self-efficacy, anxiety, pain, and vital signs (pulse and blood pressure) of patients with cancer undergoing chemotherapy. We hypothesize that relative to the control group, the patient group that receives the S-VR intervention will experience greater improvements in their comfort levels.

For peer review only

METHODS AND ANALYSIS

Design

This study is a two-arm parallel randomized controlled trial with a 1:1 allocation. The study will divide the patients into two groups: the intervention group, in which the patients will receive S-VR therapy through a VR device (head-mounted display) mounted on the patients' heads during chemotherapy plus standard care (daily routine care in administration of chemotherapy drugs), and the control group, in which the patients will receive standard care and guided imagery therapy with leaflets containing instructions for techniques for relaxing through image visualization. The group allocation will be implemented using the envelope concealment method, and the outcome assessor will be blinded to the group allocation during data collection. The protocol has been designed in accordance with the Standard Protocol Items: Recommendations for Interventional Trials and its checklist [30,31]. Figure 1 presents the modified CONSORT (2017) flow diagram [32].

Setting

The Special Region of Yogyakarta (DIY) Province will be the study location because it has the highest prevalence of cancer in Indonesia. The national prevalence of cancer is 1.79/1000 population, whereas the prevalence in the DIY Province is 4.86/1000

For peer review only

population, with 23% of these patients undergoing chemotherapy treatment [2]. This study will be conducted at the One Day Chemotherapy Polyclinic "Tulip" Integrated Cancer Unit of Dr. Sardjito General Hospital, Yogyakarta, Indonesia. According to the annual report of the service performance of the Integrated Cancer Unit of Dr. Sardjito General Hospital, the number of visits by patients with cancer was 100,614 people in 2019, an increase of 19.8% from the previous year.

Patient and public involvement

The protocol has been designed in close collaboration between the School of Nursing, Taipei Medical University, and the Master of Nursing Programme, Universitas Gadjah Mada, with strong support from Dr. Sardjito General Hospital, Yogyakarta. We work with cancer patients undergoing chemotherapy under the Integrated Cancer Unit at Dr. Sardjito General Hospital Yogyakarta, Indonesia. A patient with cancer undergoing chemotherapy was enrolled in this intervention programme and completed the study evaluation. Valuable input, preference, and evaluation on the part of both patients and hospital institutions have been invaluable in the development of this intervention, thus, we hope that the findings from this RCT will be disseminated to local, regional, and national policymakers as well as an international scientific audience.

Population and Sample

For peer review only

The target population is patients with cancer who will undergo chemotherapy at the "Tulip" Integrated Cancer Unit of Dr. Sardjito General Hospital. The inclusion criteria for this study are as follows:

1. Being a patient with cancer receiving chemotherapy in all cycles.
2. Being aged ≥18 years.
3. Having a performance status (ECOG Score) of 0–2.
4. Being able to understand and sign the informed consent form.

Respondents who meet the following exclusion criteria will be excluded from the study:

1. Being a patient with cancer with a history of skull structure or cervical spine abnormalities that may complicate the use of VR devices.
2. Being a cancer patient with cognitive, visual, and hearing impairment; or having a chemo port.
3. Being a patient with a history of epilepsy, seizures, vertigo, or visually induced motion sickness.

Sample Size Calculation

The sample size was calculated using free-to use software, namely G*Power for Windows Version 3.1.9.6 [33]. The effect size is 0.81, type 1 error (α) = 5% (two-tailed), power (1-β) 90%, and the allocation ratio between the groups is 1:1. The

For peer review only

effect size was set with reference to previous studies with similar outcomes [9]. According to the G*Power calculation, the study sample size should be 34 per group. With consideration of an expected dropout rate of 15%, the sample size was increased to 40 per group.

The participant dropout criteria are as follows:

1. The patient being unwilling to continue the sequence of the study process (lost to follow-up).
2. The patients experiencing drug extravasation incidents or severe side effects during chemotherapy.

The consecutive sampling technique will be applied. This nonprobability sampling method involves recruiting all individuals from the accessible population during a given time interval or for a specific sample size. Patients with cancer who have scheduled chemotherapy and meet the requirements of the data collection time will be asked to participate in the study.

Participant Characteristics and Study Outcomes

Participant characteristics

A questionnaire will be used to collect patient demographic data, including age, sex, education level, marital status, occupation, income, and assistance during chemotherapy, and clinical characteristics, including the type of cancer, body surface

For peer review only

area (height and weight), cancer stage, duration of diagnosis, chemotherapy cycle, and ECOG score.

Outcomes

The primary and secondary outcomes are summarized in Table 1.

Comfort of cancer patients is measured using the shortened general comfort questionnaire (SGCQ) instrument by Chatarine Kolcaba in 2006 which has been tested for validity and reliability in Indonesian version by Artanti et al. [34]. The validity test reported that Content validity index (CVI) of item (I-CVI) and scale (S-CVI) score were both 1 (valid). Cronbach's alpha score showed 0.769, meaning that the SGCQ Indonesian version is reliable. The SGCQ instrument describes individual comfort by assigning a Likert score to 28 items. Likert scores range from 1 (strongly disagree) to 6 (strongly agree). The highest total score is 168, and the lowest is 28.

Self – efficacy is measured by using the Self-Efficacy of Cancer Symptom Self-Management Scale (SE – CSSMS),[35] which consists of 6 question items with a value range of 1 (not at all sure) to 10 (very sure). SE - CSSMS has been tested for validity and reliability on cancer patients in Indonesia [35]. The content validity index (CVI) is 0.94. The r-value is 0.467, which is greater than the r-table value of 0.12, so these results state that the six items in the symptom management self-efficacy

For peer review only

instrument are valid. The reliability test results showed that the Cronbach alpha value is 0.90 (> 0.70), so the measuring instrument is reliable.

Anxiety is measured using the Visual Analog Scale for Anxiety (VAS – A) in the form of a horizontal line with a scale of 100 millimeter or 10 centimeters, with the left by the words "not at all anxious" and on the right by "extremely anxious" [36,37]. VAS-A has a sensitivity of 76.8%, specifications of 64.9%, and internal reliability consistency (Cronbach Alpha ≥0.7 [36,37].

Pain is measured using the numeric rating scale (NRS). The NRS consist of numerical scale; from 0 to 100 (101 – point scale), 0 to 10 (11 – point scale) or 1 to 10 (10 – point scale). This study will use a 0 – 10 NRS due to it provides more sufficient sensitivity to measure pain in most cancer patients.[38] The NRS has been declared valid and reliable for subjective measurement of cancer pain, both due to adverse effects of cytotoxic agents, cancer treatment procedures (surgery, radiotherapy, and chemotherapy), and the malignancy of the cancer itself [39].

Pulse rate and blood pressure (systolic and diastolic pressure) are measured using an automatic digital sphygmomanometer. According to American Heart Association, the regular resting heart rate for adults is 60 to 100 beats per minute, and systolic blood pressure is above 90 mm Hg and less than 120 mm Hg, with a diastolic pressure between 60 mm Hg and less than 80 mm Hg [40].

For peer review only

Comfort and self-efficacy for symptom management will be measured at two time points: at baseline (before chemotherapy premedication) and posttest (after the chemotherapy session is completed). Anxiety, pain, and vital signs (pulse rate and blood pressure) will be measured at five time points: thrice at pretest (baseline data collection, before the installation of intravenous access, and before administration of VR intervention) and twice at posttest (immediately after intervention and after the chemotherapy session is completed). The timeline of the data collection and measurement is presented in Table 2.

Randomization and Concealment

The researchers will screen to identify patients who are eligible to participate in the study. The patients' medical records will be evaluated to verify their baseline information (e.g., anthropometric data, laboratory data, patient identification data, and laboratory results). An allocation sequence will be generated through simple randomization of the study participants by using a formula on the Microsoft Excel program (the RANDBETWEEN function), and the allocation sequence will be concealed in sealed envelopes. Thereafter, the participants in the intervention and control groups will receive envelopes containing random numbers generated using the randomization procedure. The outcome assessor (the nurse appointed by the

For peer review only

researcher to be responsible for outcome data collection) and data analysts will be blinded to the participant allocation. Due to the technical nature of the VR interventions; it was difficult to blind the study participants. Thus, to minimize potential methodological bias, the outcome assessor and data analysts will be blinded [41].

Patient Recruitment

Recruitment will be conducted from April to July 2023. Patients who are scheduled for chemotherapy will be assessed by the nurse on duty, who will review their latest laboratory results, medical records, vital signs, and electrocardiogram results, to determine whether they are eligible to undergo chemotherapy. The patients who pass the screening process will be asked whether they are willing to be involved in the study and will be asked to sign the informed consent form. Patients who are willing and who sign the informed consent form will be given a sealed envelope containing a random number indicating their group allocation. The participants will be evaluated (pretest) by the outcome assessor (research nurse) before they enter the chemotherapy room. After the evaluation, the researcher will open each participant's envelope and see the random number that they have been assigned (without the knowledge of the participants or the outcome assessor). If the number assigned is between 1 and 40, the participant will be directed to the chemotherapy room on the east side of the hospital

For peer review only

(intervention group); if the number is between 41 and 80, the participant will be directed to the chemotherapy room on the west side of the hospital (control group). We will for the participants in the intervention and control groups to be directed to different chemotherapy rooms, and a research assistant (RA) will accompany the participants to the rooms to minimize contamination of information between participants, between participants and intervention providers and between the nurses or doctors on duty [42].

Intervention (S-VR + standard care)

Participants in the intervention arm will receive immersive VR intervention with a VR box (Shinecon 6.0 VR Box Virtual Reality Glasses with headphones). S-VR is a virtual relaxation and distraction therapy administered through a smartphone-based VR device (head-mounted display) mounted to the head of the patient with cancer during chemotherapy. The intervention involves a virtual environment in which 360° videos of natural panoramas with traditional and classical music (noncopyright) are provided. The S-VR device is paired with a smartphone with either an Android (minimum generation 11, Android 4.4 KitKat) or iOS (version 12, minimum screen size of 5.5 inch and maximum of 6.0-inch, screen resolution of at least 1080 × 1920 pixels) operating system and the ability to connect to WIFI or the Internet and with the YouTube app installed, which they will use to access the 360° videos. The S-VR

For peer review only

content comprises original 360° videos approximately 10 minutes long and produced by the researchers by using video editing software. The video duration was determined on the basis of a previous study by Fabi et al.[43] in which this duration was demonstrated to be effective in preventing patient saturation related to content and preventing motion sickness. The 360° videos present panoramic views of Yogyakarta, Indonesia, and Taipei, Taiwan (Figure 2). The RA will provide information about the S-VR intervention for approximately 5 minutes, assist in attaching the S-VR devices to the patient, and conduct the intervention for approximately 10 minutes.

Control (Leaflet + standard care)

In the control group, participants will be provided with standard care and guided imagery leaflets, which will provide information on the meaning, benefits, and methods of completing guided imagery relaxation therapy during chemotherapy. The RA will guide the participants through the practices of the guided imagery relaxation therapy described in the leaflet for ±10 minutes (Figure 3).

Compensation

Compensation will be provided by the researchers to all respondents after the chemotherapy session in the form of a package containing food, beverage, and a small

For peer review only

towel. These objects are placed in a small bag along with a note of appreciation for participation in this research.

Safety and Side Effect Management

The intervention of this study is not associated with any significant side effects because it does not involve invasive procedures and is administered for a short period (1 chemotherapy cycle). However, we predict that the participants will feel discomfort both while using the VR and during chemotherapy and will record their levels of discomfort on a monitoring sheet. The Virtual Reality Symptom Questionnaire (VRSQ), which was developed by Ames in 2005 [44], will be administered to patients after the VR intervention in order to assess the possibility of cybersickness, a form of motion sickness caused by exposure to VR. The questionnaire evaluates eight general physical side effects (general discomfort, fatigue, boredom, drowsiness, headache, dizziness, concentration difficulties, and nausea) and five visual effects (tired eyes, aching eyes, eyestrain, blurred vision, and difficulties focusing). The scoring was modified to a four-point scale (the original score consisted of a six-point scale), from 0 to 4 (represented of none, mild, moderate, severe,) with 0 indicating the absence of the symptom and 4 indicating its severity. During the study, the researchers will clearly indicate that they can be contacted should the participants have any problems or complaints or if something unexpected occurs.

For peer review only

Data Analysis

The collected data will be processed using IBM SPSS statistical software version 23 for Windows through editing, coding, and data entry processes. The statistician will be blinded to the study outcomes and will conduct analyses on the basis of the labeling of the groups. For the treatment group, the demographic and clinical variables recorded at enrollment will be tabulated. The descriptive statistics will be presented as numbers, means, standard deviations, medians, minimums, and maximums. Homogeneity and normality tests will be conducted before the bivariate analysis test. A homogeneity test for categorical data (age, sex, education level, marital status, occupation, income, the presence of a family member or caregiver during chemotherapy, type of cancer, stage of cancer, duration of cancer diagnosis, chemotherapy cycle, and ECOG scores) will be performed using the chi-square or Fisher's test, whereas a homogeneity test for numerical data (height and weight) will be performed using Levene's test if the data are normally distributed and using the Mann–Whitney U test if the data are not normally distributed. Both groups will be considered to be homogeneous if $p > 0.05$. Bivariate analysis will be used to determine the differences in the mean scores for comfort level and symptom management self-efficacy before and after treatment in the intervention and control groups. If the data distribution is normal, we will use a paired t test and independent t

For peer review only

test. However, if the data distribution is not normal, the nonparametric Wilcoxon and Mann–Whitney U tests will be used if significant differences are identified ($p < 0.05$). For anxiety, pain, and vital-sign (blood pressure and pulse rate) outcome measurement analysis, General Linear Model-Repeated Measure (GLM-RM) statistics will be used. The GLM-RM is a statistical test used for the comparative analysis of two unpaired numerical data groups with more than two measurements and is applied when the effect of a treatment or intervention is measured at different time points. If the data are not normally distributed, the repeated Mann–Whitney alternative test with Bonferroni correction will be used. The present study will use statistical method to adjust for potentially confounding effects of the S-VR intervention on the patient's outcomes. These confounders include age, gender, education, cancer stage, chemotherapy cycle, assistance during chemotherapy, and comorbidities. Multivariate model can handle identified confounders and minimize the bias effect of the intervention [45]. Linear regression analysis will be applied by using the enter method. Adjustment by using this analysis can clarify that how much the confounders in the model distort the intervention and outcome [46].

Ethics and Dissemination

The present study was approved by the Medical and Health Research Ethics Committee (MHREC) Faculty of Medicine, Public Health and Nursing Universitas

For peer review only

Gadjah Mada—Dr. Sardjito General Hospital, Yogyakarta, IRB (Approval number: KE/FK/0301/EC/2023). All participants enrolled in the study will be asked to provide written informed consent, which will include a statement requesting their willingness to allow the data to be share. All information related to the identity of the participants will be kept confidential by the researchers. No posttrial care has been planned. The study results will be published in a peer-reviewed scientific journal. The results will not indicate the identity of any participant. Authorship eligibility was based on an individual making substantial contributions to the study and manuscript review. Access to the final data set will be granted to the primary investigator and coinvestigators. The study protocol is available on clinicaltrials.gov (ClinicalTrials.gov Identifier: NCT05756465). The study is scheduled to start recruitment at Dr. Sardjito General Hospital in Indonesia in April 2023. After the implementation of the intervention, data collection, and statistical analysis, we expect to submit the results to a peer-reviewed journal in October 2023.

For peer review only

Contributors

SNG developed the initial trial concepts and drafted the manuscript. TWH and HH contributed to the development of the manuscript. All authors have critically revised the manuscript for essential intellectual content. All authors have read and approved the submitted version of the manuscript.

Funding

This work was supported by National Science and Technology Council in Taiwan grant number NSTC 111-2622-E-038 -003 for APC support.

Competing Interests

None to declare.

Patient Consent for Publication

Not required.

Provenance and Peer Review

Not commissioned; external peer review

For peer review only

REFERENCES

1 Ministry of Health Republic of Indonesia. 2019.https://www.kemkes.go.id/article/view/19020100003/hari-kanker-sedunia-2019.html

2 Basic Health Survailance/Riset Kesehatan Dasar tahun 2018. 2018. http://repository.bkpk.kemkes.go.id/3514/1/Laporan Riskesdas 2018 Nasional.pdf

3 Globocan. New Global Cancer Data: Indonesia. 2020. https://gco.iarc.fr/today/data/factsheets/populations/360-indonesia-fact-sheets.pdf

4 Thiagarajan M, Chan CMH, Fuang HG, *et al.* Symptom prevalence and related distress in cancer patients undergoing chemotherapy. *Asian Pacific Journal of Cancer Prevention* 2016;**17**:171–6.

5 Pearce A, Haas M, Viney R, *et al.* Incidence and severity of self-reported chemotherapy side effects in routine care: A prospective cohort study. *PLoS One* 2017;**12**:e0184360–e0184360.https://doi.org/10.1371/journal.pone.0184360

For peer review only

6 Şahin ZA, Ergüney S. Effect on Symptom Management Education Receiving Patients of Chemotherapy. *Journal of Cancer Education* 2016;**31**:101–7. doi:10.1007/s13187-015-0801-8

7 Society AC. Chemotherapy side effects | american cancer society. 2023.https://www.cancer.org/treatment/treatments-and-side-effects/treatment-types/chemotherapy/chemotherapy-side-effects.html

8 Keim-Malpass J, Levine B, Danhauer SC, *et al.* Work-related perceptions and quality of life among breast cancer survivors. *Psychooncology* 2016;**25**:873–6. doi:10.1002/pon.3951

9 Bilgiç Ş, Acaroğlu R. Effects of Listening to Music on the Comfort of Chemotherapy Patients. *West J Nurs Res* 2016;**39**:745–62. doi:10.1177/0193945916660527

10 Kolcaba KY. Holistic comfort: Operationalizing the construct as a nurse-sensitive outcome. *Advances in nursing science* 1992;**15**:1–10.https://www.researchgate.net/profile/Katharine-Kolcaba/publication/21635629_Holistic_comfort_operationalizing_the_construct_as_a_nurse-sensitive_outcome/links/54eb45810cf2082851bd7130/Holistic-comfort-operationalizing-the-construct-as-a-nurse-sensitive-ou

For peer review only

11 Can A, Hintistan S. An Assessment of the Comfort Level of Cancer Patients with Receiving Chemotheraphy. *Bezmialem Science* 2021;**9**:310–6. doi:10.14235/bas.galenos.2020.4226

12 Smith MC, Parker ME. *Nursing Theories and Nursing Practice 4th ed.* Philadelphia, PA: : F.A. Davis 2015.

13 Kolcaba KY. The Art of Comfort Care. *Image: the Journal of Nursing Scholarship* 1995;**27**:287–9. doi:https://doi.org/10.1111/j.1547-5069.1995.tb00889.x

14 Nuraini T, Gayatri D, Rachmawati İ. Comfort assessment of cancer patient in palliative care: A nursing perspective. *Int J Caring Sci* 2017;**10**:209.https://internationaljournalofcaringsciences.org/docs/24_tuti_original_10_1.pdf

15 Kolcaba K. *Comfort Theory and Practice: A Vision for Holistic Health Care and Research*. New York, NY: : Springer Publishing Company 2003.

16 Cheng F, Wang W. Factors influencing comfort level in head and neck neoplasm patients receiving radiotherapy. *Int J Nurs Sci* 2014;**1**:394–9. doi:https://doi.org/10.1016/j.ijnss.2014.10.009

17 Miao J, Liu M, Ma J, *et al.* Effectiveness of Comfort Nursing Combined with Continuous Nursing on Patients with Colorectal Cancer Chemotherapy.

For peer review only

Evidence-Based Complementary and Alternative Medicine 2022;**2022**:9647325. doi:10.1155/2022/9647325

18 Prigerson HG, Bao Y, Shah MA, *et al.* Chemotherapy Use, Performance Status, and Quality of Life at the End of Life. *JAMA Oncol* 2015;**1**:778–84. doi:10.1001/jamaoncol.2015.2378

19 Bani Mohammad E, Ahmad M. Virtual reality as a distraction technique for pain and anxiety among patients with breast cancer: A randomized control trial. *Palliat Support Care* 2019;**17**:29–34. doi:DOI: 10.1017/S1478951518000639

20 Scates D, Dickinson JI, Sullivan K, *et al.* Using Nature-Inspired Virtual Reality as a Distraction to Reduce Stress and Pain Among Cancer Patients. *Environ Behav* 2020;**52**:895–918. doi:10.1177/0013916520916259

21 Chirico A, Lucidi F, De Laurentiis M, *et al.* Virtual Reality in Health System: Beyond Entertainment. A Mini-Review on the Efficacy of VR During Cancer Treatment. *J Cell Physiol* 2016;**231**:275–87. doi:https://doi.org/10.1002/jcp.25117

22 Chirico A, Maiorano P, Indovina P, *et al.* Virtual reality and music therapy as distraction interventions to alleviate anxiety and improve mood states in breast cancer patients during chemotherapy. *J Cell Physiol* 2020;**235**:5353–62. doi:https://doi.org/10.1002/jcp.29422

For peer review only

23 Stetz MAJ, Ries RI, Folen RA. Virtual reality supporting psychological health. In: *Advanced Computational Intelligence Paradigms in Healthcare 6. Virtual Reality in Psychotherapy, Rehabilitation, and Assessment*. Springer 2011. 13–29.

24 Morina N, Ijntema H, Meyerbröker K, *et al.* Can virtual reality exposure therapy gains be generalized to real-life? A meta-analysis of studies applying behavioral assessments. *Behaviour Research and Therapy* 2015;**74**:18–24. doi:https://doi.org/10.1016/j.brat.2015.08.010

25 Rubio-Tamayo JL, Barrio MG, García FG. Immersive Environments and Virtual Reality: Systematic Review and Advances in Communication, Interaction and Simulation. *Multimodal Technologies and Interaction 2017, Vol 1, Page 21* 2017;**1**:21. doi:10.3390/MTI1040021

26 Boeldt D, McMahon E, McFaul M, *et al.* Using Virtual Reality Exposure Therapy to Enhance Treatment of Anxiety Disorders: Identifying Areas of Clinical Adoption and Potential Obstacles. *Front Psychiatry* 2019;**10**:476694. doi:10.3389/FPSYT.2019.00773/BIBTEX

27 Huang Q, Lin J, Han R, *et al.* Using Virtual Reality Exposure Therapy in Pain Management: A Systematic Review and Meta-Analysis of Randomized

For peer review only

Controlled Trials. *Value in Health* 2022;**25**:288–301. doi:https://doi.org/10.1016/j.jval.2021.04.1285

28 Groninger H, Stewart D, Wesley D, *et al.* Virtual reality for management of cancer pain: Study rationale and design. *Contemp Clin Trials Commun* 2022;**26**:100895. doi:https://doi.org/10.1016/j.conctc.2022.100895

29 Rutkowski S, Czech O, Wrzeciono A, *et al.* Virtual reality as a chemotherapy support in treatment of anxiety and fatigue in patients with cancer: A systematic review and meta-analysis and future research directions. *Complement Ther Med* 2021;**61**:102767. doi:https://doi.org/10.1016/j.ctim.2021.102767

30 Chan A-W, Tetzlaff JM, Gøtzsche PC, *et al.* SPIRIT 2013 explanation and elaboration: guidance for protocols of clinical trials. *BMJ : British Medical Journal* 2013;**346**:e7586–e7586. doi:10.1136/bmj.e7586

31 Calvert M, King M, Mercieca-Bebber R, *et al.* SPIRIT-PRO Extension explanation and elaboration: guidelines for inclusion of patient-reported outcomes in protocols of clinical trials. *BMJ Open* 2021;**11**:e045105–e045105. doi:10.1136/bmjopen-2020-045105

32 Boutron I, Altman DG, Moher D, *et al.* CONSORT Statement for Randomized Trials of Nonpharmacologic Treatments: A 2017 Update and a CONSORT

For peer review only

Extension for Nonpharmacologic Trial Abstracts. *Ann Intern Med* 2017;**167**:40–7. doi:10.7326/M17-0046

33 Faul F, Erdfelder E, Buchner A, *et al.* Statistical power analyses using G*Power 3.1: Tests for correlation and regression analyses. *Behav Res Methods* 2009;**41**:1149–60. doi:10.3758/BRM.41.4.1149

34 Artanti ER, Nurjannah I, Subroto S. Validity and reliability of shortened general Comfort questionnaire in indonesian version. *Belitung Nurs J* 2018;**4**:366–72. doi:10.33546/bnj.437

35 Mumtaz DF, Effendy C, Haryani H. Impact of Pre-Chemotherapy Education with Audio Visual Methods on the Self-Efficacy of Symptom Management in Patients with Cancer. *Journal of Cancer Education* 2022;**37**:1546–52. doi:10.1007/s13187-021-02006-1

36 Davey HM, Barratt AL, Butow PN, *et al.* A one-item question with a Likert or Visual Analog Scale adequately measured current anxiety. *J Clin Epidemiol* 2007;**60**:356–60. doi:https://doi.org/10.1016/j.jclinepi.2006.07.015

37 Facco E, Zanette G, Favero L, *et al.* Toward the Validation of Visual Analogue Scale for Anxiety. *Anesth Prog* 2011;**58**:8–13. doi:10.2344/0003-3006-58.1.8

38 Paice JA, Cohen FL. Validity of a verbally administered numeric rating scale to measure cancer pain intensity. *Cancer Nurs*

For peer review only

1997;**20**.https://journals.lww.com/cancernursingonline/Fulltext/1997/04000/Validity_of_a_verbally_administered_numeric_rating.2.aspx

39 Brunelli C, Zecca E, Martini C, *et al.* Comparison of numerical and verbal rating scales to measure pain exacerbations in patients with chronic cancer pain. *Health Qual Life Outcomes* 2010;**8**. doi:10.1186/1477-7525-8-42

40 American Heart Association | To be a relentless force for a world of longer, healthier lives. https://www.heart.org/en/ (accessed 17 Jun 2023).

41 Ahmad M, Bani Mohammad E, Anshasi HA. Virtual Reality Technology for Pain and Anxiety Management among Patients with Cancer: A Systematic Review. *Pain Management Nursing* 2020;**21**:601–7. doi:https://doi.org/10.1016/j.pmn.2020.04.002

42 Magill N, Knight R, McCrone P, *et al.* A scoping review of the problems and solutions associated with contamination in trials of complex interventions in mental health. *BMC Med Res Methodol* 2019;**19**:4. doi:10.1186/s12874-018-0646-z

43 Fabi A, Fotia L, Giuseppini F, *et al.* The immersive experience of virtual reality during chemotherapy in patients with early breast and ovarian cancers: the Patient's Dream study. *Front Oncol* 2022;:4271.

For peer review only

44 Ames SL, Wolffsohn JS, Mcbrien NA. The development of a symptom questionnaire for assessing virtual reality viewing using a head-mounted display. *Optom Vis Sci* 2005;**82**:168–76. doi:10.1097/01.OPX.0000156307.95086.6

45 McNamee R. Regression modelling and other methods to control confounding. *Occup Environ Med* 2005;**62**:500 LP – 506. doi:10.1136/oem.2002.001115

46 Pourhoseingholi MA, Baghestani AR, Vahedi M. How to control confounding effects by statistical analysis. *Gastroenterol Hepatol Bed Bench* 2012;**5**:79./pmc/articles/PMC4017459/ (accessed 17 Jun 2023).

For peer review only

Table and Figure Legends

Figure 1. Modified CONSORT Flow Diagram

Figure 2. 360° videos used in the S-VR intervention

Figure 3. Guided imagery leaflet

Table 1. Study outcomes

Table 2. Timeline of data collection and measurements

For peer review only

Table 1. Study outcomes

Outcome	Measurement tool	Score	Interpretation
**Primary**			
Comfort	the Shortened General Comfort Questionnaire, Indonesian version (SGCQ) (41)	Likert scale—1 (strongly disagree) to 6 (strongly agree)	Higher scores indicate higher levels of comfort.
**Secondary**			
Symptom management self-efficacy	the Self-Efficacy of Cancer Symptom Self-Management Scale (SE-CSSMS) (42)	Likert scale—1 (not at all sure) to 10 (very sure)	Higher scores indicate higher levels of self-efficacy.
Anxiety	the Visual Analog Scale for Anxiety (VAS-A) (43,44)	A horizontal line with a scale of 100 mm	Higher scores indicate greater anxiety.
Pain	the Numerical Rating Pain Scale (NPS) (45)	Numerical scale—0 to 10 (11-point scale)	Higher scores indicate greater pain.
Pulse rate	Digital sphygmomanometer	Beats per minute (bpm)	An adult's regular resting heart rate ranges from 60 to 100 bpm.
Blood pressure	Digital sphygmomanometer	Millimeter of mercury (mmHg)	Systolic pressure above 90 mmHg and lower than 120 mmHg and diastolic pressure between 60 mmHg and 80 mmHg

For peer review only

Table 2. Timeline of data collection and measurements

Assessment/measurement instrument	Study period				
	T0	T1	T2	T3	T4
Demographic variables	**X**				
Clinical characteristics	**X**				
SGCQ	**X**				**X**
SEQSM	**X**				**X**
VAS-A	**X**	**X**	**X**	**X**	**X**
NPS	**X**	**X**	**X**	**X**	**X**
Blood pressure	**X**	**X**	**X**	**X**	**X**
Pulse rate	**X**	**X**	**X**	**X**	**X**

T0: Baseline data collection (after prechemotherapy assessment and obtaining signed informed consent); T1: Before intravenous installation/premedication; T2: Before the start of the intervention; T3: Immediately after the intervention; T4: After completion of one chemotherapy cycle

For peer review only

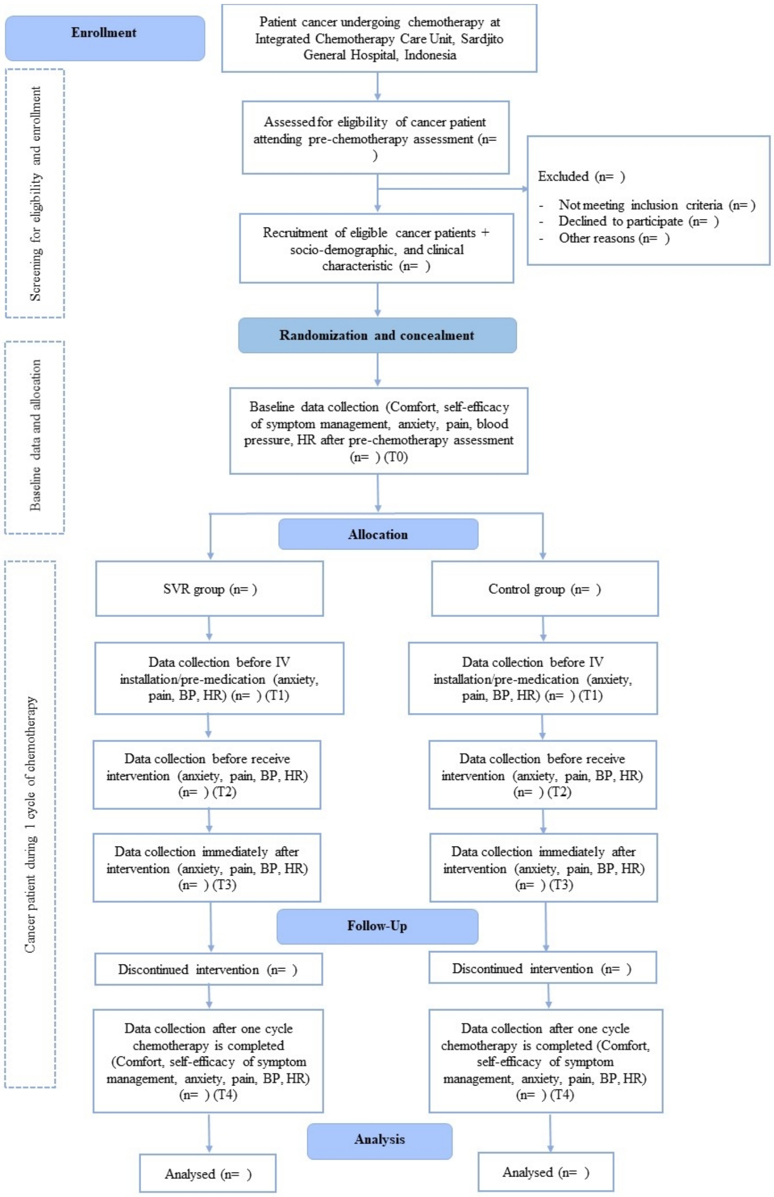

Figure 1. Modified CONSORT Flow Diagram

85x113mm (300 x 300 DPI)

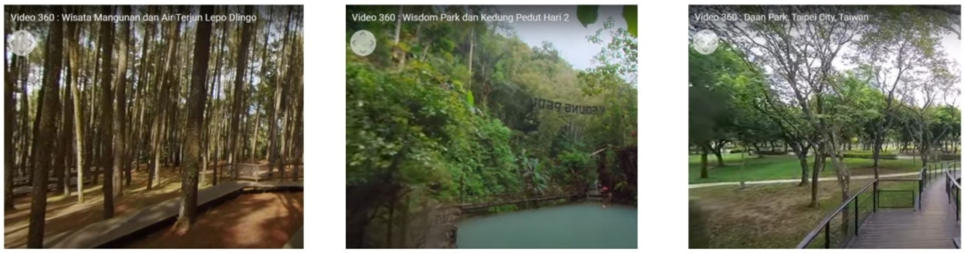

360° Video: Pine Forest and Waterfall in Yogyakarta, Indonesia (https://www.youtube.com/watch?v=OJ5LpBqiRk8)

360° Video: Wisdom Park and Natural Pond in Yogyakarta, Indonesia (https://www.youtube.com/watch?v=6A1RTps5fdY)

360° Video: Daan Park in Taipei City, Taiwan (https://www.youtube.com/watch?v=-h8JgPONKws

Figure 2. 360° videos used in the S-VR intervention

174x87mm (300 x 300 DPI)

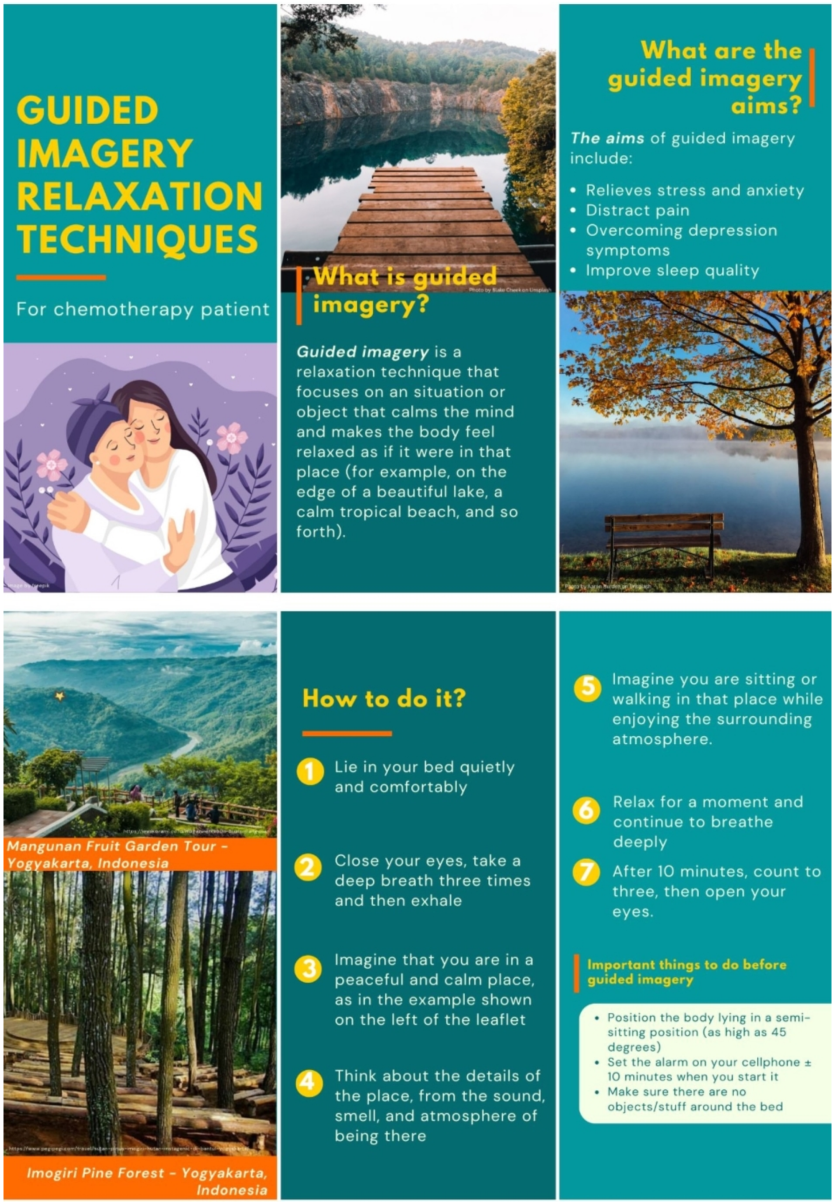

Figure 3. Guided imagery leaflet

133x192mm (300 x 300 DPI)

CONSORT 2010 checklist of information to include when reporting a randomised trial*

Efficacy of Smartphone-Based Virtual Reality Relaxation in Providing Comfort to Patients with Cancer Undergoing Chemotherapy: Protocol for a Randomized Controlled Trial

Section/Topic	Item No	Checklist item	Reported on page No
**Title and abstract**			
	1a	Identification as a randomised trial in the title	1
	1b	Structured summary of trial design, methods, results, and conclusions (for specific guidance see CONSORT for abstracts)	4 - 5
**Introduction**			
Background and objectives	2a	Scientific background and explanation of rationale	6 – 8
	2b	Specific objectives or hypotheses	8
**Methods**			
Trial design	3a	Description of trial design (such as parallel, factorial) including allocation ratio	9
	3b	Important changes to methods after trial commencement (such as eligibility criteria), with reasons	10 – 11
Participants	4a	Eligibility criteria for participants	10 – 11
	4b	Settings and locations where the data were collected	9
Interventions	5	The interventions for each group with sufficient details to allow replication, including how and when they were actually administered	14 – 15
Outcomes	6a	Completely defined pre-specified primary and secondary outcome measures, including how and when they were assessed	11 – 13
	6b	Any changes to trial outcomes after the trial commenced, with reasons	NA
Sample size	7a	How sample size was determined	10 – 11
	7b	When applicable, explanation of any interim analyses and stopping guidelines	NA
Randomisation:			
Sequence generation	8a	Method used to generate the random allocation sequence	13 – 14
	8b	Type of randomisation; details of any restriction (such as blocking and block size)	13 – 14
Allocation concealment mechanism	9	Mechanism used to implement the random allocation sequence (such as sequentially numbered containers), describing any steps taken to conceal the sequence until interventions were assigned	13 – 14
Implementation	10	Who generated the random allocation sequence, who enrolled participants, and who assigned participants to	13 – 14

For peer review only

For peer review only - http://bmjopen.bmj.com/site/about/guidelines.xhtml

		interventions	
Blinding	11a	If done, who was blinded after assignment to interventions (for example, participants, care providers, those assessing outcomes) and how	13 – 14
	11b	If relevant, description of the similarity of interventions	15
Statistical methods	12a	Statistical methods used to compare groups for primary and secondary outcomes	16 – 17
	12b	Methods for additional analyses, such as subgroup analyses and adjusted analyses	17
**Results**			
Participant flow (a diagram is strongly recommended)	13a	For each group, the numbers of participants who were randomly assigned, received intended treatment, and were analysed for the primary outcome	Figure 1
	13b	For each group, losses and exclusions after randomisation, together with reasons	NA
Recruitment	14a	Dates defining the periods of recruitment and follow-up	NA
	14b	Why the trial ended or was stopped	NA
Baseline data	15	A table showing baseline demographic and clinical characteristics for each group	NA
Numbers analysed	16	For each group, number of participants (denominator) included in each analysis and whether the analysis was by original assigned groups	NA
Outcomes and estimation	17a	For each primary and secondary outcome, results for each group, and the estimated effect size and its precision (such as 95% confidence interval)	NA
	17b	For binary outcomes, presentation of both absolute and relative effect sizes is recommended	NA
Ancillary analyses	18	Results of any other analyses performed, including subgroup analyses and adjusted analyses, distinguishing pre-specified from exploratory	NA
Harms	19	All important harms or unintended effects in each group (for specific guidance see CONSORT for harms)	NA
**Discussion**			
Limitations	20	Trial limitations, addressing sources of potential bias, imprecision, and, if relevant, multiplicity of analyses	NA
Generalisability	21	Generalisability (external validity, applicability) of the trial findings	NA
Interpretation	22	Interpretation consistent with results, balancing benefits and harms, and considering other relevant evidence	NA
**Other information**			
Registration	23	Registration number and name of trial registry	2 & 18
Protocol	24	Where the full trial protocol can be accessed, if available	18
Funding	25	Sources of funding and other support (such as supply of drugs), role of funders	18 – 19

For peer review only

For peer review only - http://bmjopen.bmj.com/site/about/guidelines.xhtml

*We strongly recommend reading this statement in conjunction with the CONSORT 2010 Explanation and Elaboration for important clarifications on all the items. If relevant, we also recommend reading CONSORT extensions for cluster randomised trials, non-inferiority and equivalence trials, non-pharmacological treatments, herbal interventions, and pragmatic trials. Additional extensions are forthcoming: for those and for up to date references relevant to this checklist, see www.consort-statement.org.

For peer review only

SPIRIT
STANDARD PROTOCOL ITEMS: RECOMMENDATIONS FOR INTERVENTIONAL TRIALS

SPIRIT 2013 Checklist: Recommended items to address in a clinical trial protocol and related documents*

Section/item	Item No	Description	Pages
**Administrative information**			
Title	1	Descriptive title identifying the study design, population, interventions, and, if applicable, trial acronym	1
Trial registration	2a	Trial identifier and registry name. If not yet registered, name of intended registry	4 – 5
	2b	All items from the World Health Organization Trial Registration Data Set	NA
Protocol version	3	Date and version identifier	2
Funding	4	Sources and types of financial, material, and other support	3
Roles and responsibilities	5a	Names, affiliations, and roles of protocol contributors	3
	5b	Name and contact information for the trial sponsor	NA
	5c	Role of study sponsor and funders, if any, in study design; collection, management, analysis, and interpretation of data; writing of the report; and the decision to submit the report for publication, including whether they will have ultimate authority over any of these activities	NA
	5d	Composition, roles, and responsibilities of the coordinating centre, steering committee, endpoint adjudication committee, data management team, and other individuals or groups overseeing the trial, if applicable (see Item 21a for data monitoring committee)	NA
**Introduction**			
Background and rationale	6a	Description of research question and justification for undertaking the trial, including summary of relevant studies (published and unpublished) examining benefits and harms for each intervention	8 – 9
	6b	Explanation for choice of comparators	8 – 9
Objectives	7	Specific objectives or hypotheses	10

For peer review only - http://bmjopen.bmj.com/site/about/guidelines.xhtml

Trial design	8	Description of trial design including type of trial (eg, parallel group, crossover, factorial, single group), allocation ratio, and framework (eg, superiority, equivalence, noninferiority, exploratory)	10
**Methods: Participants, interventions, and outcomes**			
Study setting	9	Description of study settings (eg, community clinic, academic hospital) and list of countries where data will be collected. Reference to where list of study sites can be obtained	11
Eligibility criteria	10	Inclusion and exclusion criteria for participants. If applicable, eligibility criteria for study centres and individuals who will perform the interventions (eg, surgeons, psychotherapists)	13 – 14
Interventions	11a	Interventions for each group with sufficient detail to allow replication, including how and when they will be administered	19 – 20
	11b	Criteria for discontinuing or modifying allocated interventions for a given trial participant (eg, drug dose change in response to harms, participant request, or improving/worsening disease)	13
	11c	Strategies to improve adherence to intervention protocols, and any procedures for monitoring adherence (eg, drug tablet return, laboratory tests)	NA
	11d	Relevant concomitant care and interventions that are permitted or prohibited during the trial	NA
Outcomes	12	Primary, secondary, and other outcomes, including the specific measurement variable (eg, systolic blood pressure), analysis metric (eg, change from baseline, final value, time to event), method of aggregation (eg, median, proportion), and time point for each outcome. Explanation of the clinical relevance of chosen efficacy and harm outcomes is strongly recommended	15 – 17
Participant timeline	13	Time schedule of enrolment, interventions (including any run-ins and washouts), assessments, and visits for participants. A schematic diagram is highly recommended (see Figure)	Table 2
Sample size	14	Estimated number of participants needed to achieve study objectives and how it was determined, including clinical and statistical assumptions supporting any sample size calculations	13-14

Recruitment	15	Strategies for achieving adequate participant enrolment to reach target sample size	13-14
**Methods: Assignment of interventions (for controlled trials)**			
Allocation:			
Sequence generation	16a	Method of generating the allocation sequence (eg, computer-generated random numbers), and list of any factors for stratification. To reduce predictability of a random sequence, details of any planned restriction (eg, blocking) should be provided in a separate document that is unavailable to those who enrol participants or assign interventions	20
Allocation concealment mechanism	16b	Mechanism of implementing the allocation sequence (eg, central telephone; sequentially numbered, opaque, sealed envelopes), describing any steps to conceal the sequence until interventions are assigned	17
Implementation	16c	Who will generate the allocation sequence, who will enrol participants, and who will assign participants to interventions	17-18
Blinding (masking)	17a	Who will be blinded after assignment to interventions (eg, trial participants, care providers, outcome assessors, data analysts), and how	17-18
	17b	If blinded, circumstances under which unblinding is permissible, and procedure for revealing a participant's allocated intervention during the trial	17-18
**Methods: Data collection, management, and analysis**			
Data collection methods	18a	Plans for assessment and collection of outcome, baseline, and other trial data, including any related processes to promote data quality (eg, duplicate measurements, training of assessors) and a description of study instruments (eg, questionnaires, laboratory tests) along with their reliability and validity, if known. Reference to where data collection forms can be found, if not in the protocol	12-17
	18b	Plans to promote participant retention and complete follow-up, including list of any outcome data to be collected for participants who discontinue or deviate from intervention protocols	NA
Data management	19	Plans for data entry, coding, security, and storage, including any related processes to promote data quality (eg, double data entry; range checks for data values). Reference to where details of data management procedures can be found, if not in the protocol	NA

Statistical methods	20a	Statistical methods for analysing primary and secondary outcomes. Reference to where other details of the statistical analysis plan can be found, if not in the protocol	22-23
	20b	Methods for any additional analyses (eg, subgroup and adjusted analyses)	22-23
	20c	Definition of analysis population relating to protocol non-adherence (eg, as randomised analysis), and any statistical methods to handle missing data (eg, multiple imputation)	NA
**Methods: Monitoring**			
Data monitoring	21a	Composition of data monitoring committee (DMC); summary of its role and reporting structure; statement of whether it is independent from the sponsor and competing interests; and reference to where further details about its charter can be found, if not in the protocol. Alternatively, an explanation of why a DMC is not needed	NA
	21b	Description of any interim analyses and stopping guidelines, including who will have access to these interim results and make the final decision to terminate the trial	NA
Harms	22	Plans for collecting, assessing, reporting, and managing solicited and spontaneously reported adverse events and other unintended effects of trial interventions or trial conduct	21
Auditing	23	Frequency and procedures for auditing trial conduct, if any, and whether the process will be independent from investigators and the sponsor	NA
**Ethics and dissemination**			
Research ethics approval	24	Plans for seeking research ethics committee/institutional review board (REC/IRB) approval	23-24
Protocol amendments	25	Plans for communicating important protocol modifications (eg, changes to eligibility criteria, outcomes, analyses) to relevant parties (eg, investigators, REC/IRBs, trial participants, trial registries, journals, regulators)	NA
Consent or assent	26a	Who will obtain informed consent or assent from potential trial participants or authorised surrogates, and how (see Item 32)	13
	26b	Additional consent provisions for collection and use of participant data and biological specimens in ancillary studies, if applicable	NA

only

For peer review only - http://bmjopen.bmj.com/site/about/guidelines.xhtml

Confidentiality	27	How personal information about potential and enrolled participants will be collected, shared, and maintained in order to protect confidentiality before, during, and after the trial	22-24
Declaration of interests	28	Financial and other competing interests for principal investigators for the overall trial and each study site	25
Access to data	29	Statement of who will have access to the final trial dataset, and disclosure of contractual agreements that limit such access for investigators	NA
Ancillary and post-trial care	30	Provisions, if any, for ancillary and post-trial care, and for compensation to those who suffer harm from trial participation	NA
Dissemination policy	31a	Plans for investigators and sponsor to communicate trial results to participants, healthcare professionals, the public, and other relevant groups (eg, via publication, reporting in results databases, or other data sharing arrangements), including any publication restrictions	23-24
	31b	Authorship eligibility guidelines and any intended use of professional writers	NA
	31c	Plans, if any, for granting public access to the full protocol, participant-level dataset, and statistical code	NA
**Appendices**			
Informed consent materials	32	Model consent form and other related documentation given to participants and authorised surrogates	NA
Biological specimens	33	Plans for collection, laboratory evaluation, and storage of biological specimens for genetic or molecular analysis in the current trial and for future use in ancillary studies, if applicable	NA

*It is strongly recommended that this checklist be read in conjunction with the SPIRIT 2013 Explanation & Elaboration for important clarification on the items. Amendments to the protocol should be tracked and dated. The SPIRIT checklist is copyrighted by the SPIRIT Group under the Creative Commons "Attribution-NonCommercial-NoDerivs 3.0 Unported" license.

For peer review only
